# Enhancer Activation by Pharmacologic Displacement of LSD1 from GFI1 Induces Differentiation in Acute Myeloid Leukemia

**DOI:** 10.1016/j.celrep.2018.03.012

**Published:** 2018-03-27

**Authors:** Alba Maiques-Diaz, Gary J. Spencer, James T. Lynch, Filippo Ciceri, Emma L. Williams, Fabio M.R. Amaral, Daniel H. Wiseman, William J. Harris, Yaoyong Li, Sudhakar Sahoo, James R. Hitchin, Daniel P. Mould, Emma E. Fairweather, Bohdan Waszkowycz, Allan M. Jordan, Duncan L. Smith, Tim C.P. Somervaille

**Affiliations:** 1 Leukaemia Biology Laboratory, Cancer Research UK Manchester Institute, The University of Manchester, Manchester Cancer Research Centre Building, 555 Wilmslow Road, Manchester M20 4GJ, UK; 2 Computational Biology Support, Cancer Research UK Manchester Institute, The University of Manchester, Manchester Cancer Research Centre Building, 555 Wilmslow Road, Manchester M20 4GJ, UK; 3 Drug Discovery Unit, Cancer Research UK Manchester Institute, The University of Manchester, Manchester Cancer Research Centre Building, 555 Wilmslow Road, Manchester M20 4GJ, UK; 4 Biological Mass Spectrometry Facility, Cancer Research UK Manchester Institute, The University of Manchester, Manchester Cancer Research Centre Building, 555 Wilmslow Road, Manchester M20 4GJ, UK

**Keywords:** LSD1, GFI1, acute myeloid leukemia, MLL, acetylation, methylation

## Abstract

Pharmacologic inhibition of LSD1 promotes blast cell differentiation in acute myeloid leukemia (AML) with *MLL* translocations. The assumption has been that differentiation is induced through blockade of LSD1’s histone demethylase activity. However, we observed that rapid, extensive, drug-induced changes in transcription occurred without genome-wide accumulation of the histone modifications targeted for demethylation by LSD1 at sites of LSD1 binding and that a demethylase-defective mutant rescued *LSD1* knockdown AML cells as efficiently as wild-type protein. Rather, LSD1 inhibitors disrupt the interaction of LSD1 and RCOR1 with the SNAG-domain transcription repressor GFI1, which is bound to a discrete set of enhancers located close to transcription factor genes that regulate myeloid differentiation. Physical separation of LSD1/RCOR1 from GFI1 is required for drug-induced differentiation. The consequent inactivation of GFI1 leads to increased enhancer histone acetylation within hours, which directly correlates with the upregulation of nearby subordinate genes.

## Highlights

• Inhibitors of LSD1 target both scaffolding and enzymatic functions of the protein
• GFI1/CoREST complex is targeted for disruption and release from chromatin
• GFI1/CoREST disruption is required for leukemia cell differentiation
• Loss of enhancer-bound GFI1/LSD1 activates nearby myeloid differentiation genes

## Graphical Abstract

**Figure:**
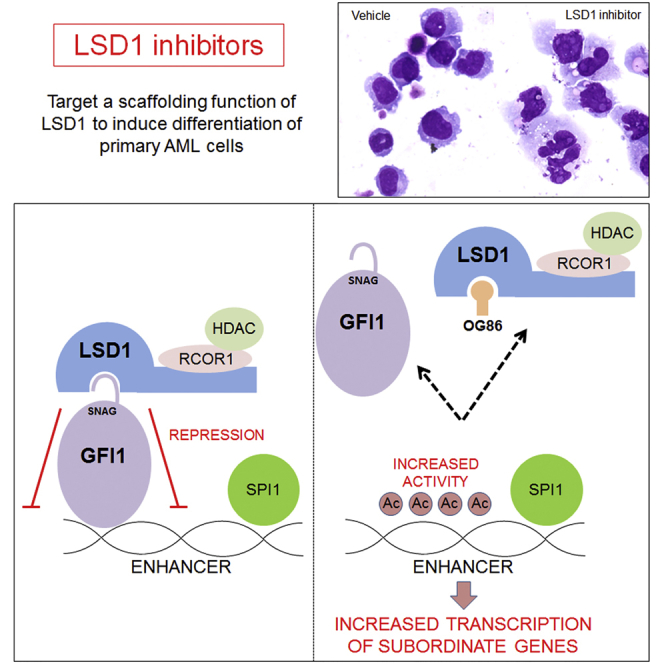

## Teaser

Maiques-Diaz et al. report that, while LSD1 inhibitors target both scaffolding and enzymatic functions of the protein, drug-induced myeloid leukemia cell differentiation is primarily due to the disruption and release from enhancers of GFI1/CoREST complexes, leading to the activation of subordinate myeloid transcription factor genes.

## Introduction

Lysine-specific demethylase 1 (LSD1, also known as KDM1A, AOF2, BHC110 or KIAA0601) is one of a number of epigenetic regulators that have recently emerged as candidate therapeutic targets in cancer. It was initially identified as a core component of an RCOR1 (CoREST) histone deacetylase (HDAC) transcription corepressor complex (You et al., 2001) and later found to have lysine-specific demethylase activity (Shi et al., 2004). With regard to its enzymatic function, LSD1 is a flavin adenine dinucleotide (FAD)-dependent homolog of the amine oxidase family, with an ability to demethylate monomethyl or dimethyl lysine 4 (K4) of histone H3, releasing hydrogen peroxide and formaldehyde (Shi et al., 2004). Its interaction through its Tower domain with RCOR1, or MTA2 when part of the NuRD complex, is required for demethylation of nucleosomes (Lee et al., 2005, Shi et al., 2005, Wang et al., 2009). In addition to H3 K4, LSD1 has also been reported to demethylate other lysine targets such as H3 K9, DNMT1, and TP53 to functional effect (Lynch et al., 2012).

The interest in LSD1 as a therapeutic target in cancer arose from the observation of its high-level expression in poor prognosis sub-groups of prostate, lung, brain, and breast cancer, as well as in certain hematologic malignancies (Maiques-Diaz & Somervaille, 2016). The first drug found to inhibit LSD1 was tranylcypromine (TCP), a monoamine oxidase inhibitor used in the treatment of depression (Lee et al., 2006b). TCP is a mechanism-based suicide inactivator of LSD1 that covalently attaches to the N(5) and C(4a) residues of the isoalloxazine ring of FAD, which is itself located deep within the active site of LSD1 (Schmidt and McCafferty, 2007, Yang et al., 2007). To improve the potency and selectivity of TCP toward LSD1, derivatives active in the nanomolar range have been developed (Guibourt et al., 2010, Johnson and Kasparec, 2012, Maiques-Diaz and Somervaille, 2016), and these have shown significant promise as differentiation-inducing agents in pre-clinical studies in acute myeloid leukemia (AML) (Harris et al., 2012, Schenk et al., 2012). With LSD1 inhibitors advancing through early-phase clinical trials, an appreciation of their mechanism of action is essential. The assumption has been that LSD1 contributes to gene repression by removing monomethyl and dimethyl histone marks from lysine 4 of histone H3 and that this is the key activity targeted for potential therapeutic effect. However, LSD1 also interacts with multiple transcription factors (Lynch et al., 2012), raising the possibility that other mechanisms may be significant.

## Results

### OG86 Induces a Myeloid Differentiation Program in MLL-Translocated AML Cells

**Figure 1:**
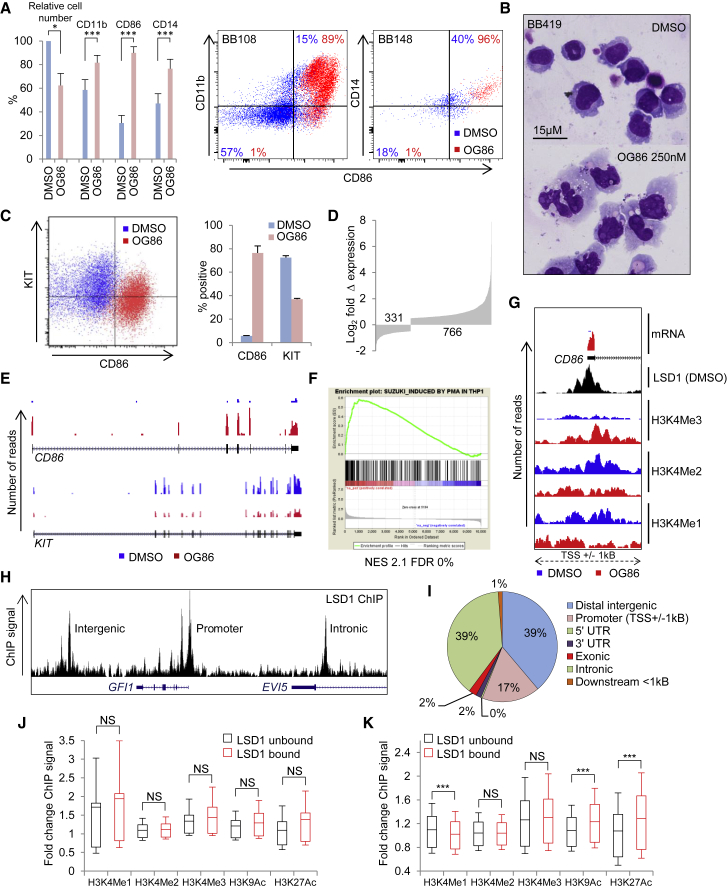
Absence of H3K4Me1 or H3K4Me2 Accumulation at Active Enhancers and Upregulated Promoters following LSD1 Inhibition In (A) and (B), primary patient AML cells with MLL translocations (n = 8 separate cases) were treated for 7 days in stromal co-culture with 250 nM OG86 or DMSO vehicle. (A) Bar graph and exemplar fluorescence-activated cell sorting (FACS) plots show changes in relative cell numbers and immunophenotype; ^∗^p < 0.05, ^∗∗∗^p < 0.005, t test. (B) Exemplar cytospin preparations. In (C)–(K), THP1 AML cells were treated for 24 hr with 250 nM OG86 or DMSO vehicle. (C) Exemplar flow cytometry plot (left) and bar graph (right) indicate expression of CD86 and KIT. Error bars indicate SEM (n = 4). (D) Numbers of up- and downregulated genes. (E) Exemplar RNA-seq tracks. (F) Gene set enrichment analysis plot. NES, normalized enrichment score; FDR, false discovery rate. (G) Exemplar RNA-seq and ChIP-seq tracks. (H) Exemplar LSD1 ChIPseq track. (I) Pie chart indicates genome location annotations for 18,937 LSD1 binding peaks. (J and K) Box-and-whisker plots show mean, 20^th^, and 80^th^ percentile values (box) and 10^th^ and 90^th^ percentile values (whiskers) for fold change in ChIP signal for the indicated histone marks at (J) promoter regions (TSS ± 2.5 kb) of upregulated genes or (K) active intergenic enhancers (enhancer center ± 2.5 kb), according to LSD1 binding status. ^∗∗∗^p < 0.001, t test; NS, not significant. See also Figure S1 and Tables S1, S2, and S3.

Treatment of patients with *MLL*-translocated AML with ORY1001, a tranylcypromine-derivative inhibitor of LSD1 (Maes et al., 2015), induces differentiation of blast cells in blood and bone marrow (Somervaille et al., 2016); and the related potent and selective inhibitor trans-N-((2-methoxypyridin-3-yl)methyl)-2-phenylcyclopropan-1-amine (hereinafter termed OG86, for Oryzon Genomics compound 86) impairs proliferation and induces differentiation of primary *MLL*-translocated AML blast cells *in vitro*, as evidenced by both upregulation of immunophenotypic markers of myeloid differentiation and morphology (Figures 1A and 1B; Table S1; Harris et al., 2012). If the primary mechanism by which these compounds induce differentiation is through blockade of the histone demethylase activity of LSD1, it would be expected that changes in transcription due to LSD1 inhibition (which are detected within 1 hr; Lynch et al., 2013) would be tightly correlated with co-localized increases in mono- and dimethyl histone H3K4 methylation, the modifications targeted for demethylation by LSD1. To determine whether this is the case, we treated THP1 AML cells with OG86 or DMSO vehicle for 24 hr and then performed concomitant RNA sequencing (RNA-seq) and chromatin immunoprecipitation with next-generation sequencing (ChIP-seq) using antibodies against histone methylation and acetylation marks, as well as LSD1. THP1 cells exhibit a t(9;11) *MLL* gene rearrangement and display similar phenotypic responses following LSD1 inhibition to those observed in primary *MLL*-translocated AML cells (Figures 1A–1C; Table S2).

Regarding the transcriptome, and focusing on the 10,002 expressed protein-coding genes (Table S2), there were extensive changes in transcription in OG86-treated cells with 766 and 331 genes exhibiting log_2_ fold changes in expression of 0.5 and −0.5, respectively (Figure 1D; Table S2). Among the most highly upregulated genes was *CD86*, which is induced during monocyte/macrophage differentiation (Lynch et al., 2013), and among the most highly downregulated was *KIT*, which is expressed by hematopoietic stem and progenitor cells (HSPCs) and downregulated during differentiation (Figure 1E; Table S2). Concomitant protein changes were confirmed by flow cytometry (Figure 1C). Comparison of the transcription changes induced by LSD1 inhibition with those observed during phorbol-ester-mediated terminal differentiation of THP1 AML cells into macrophages (Suzuki et al., 2009) revealed a highly significant overlap (Figure 1F). Thus, pharmacologic inhibition of LSD1 induces substantial upregulation of a myeloid differentiation transcription program within 24 hr, with morphologic differentiation ensuing thereafter (Figure 1B; Harris et al., 2012).

### Lack of Selective Accumulation of H3K4Me1/2 at LSD1-Bound Promoters and Active Enhancers following LSD1 Inhibition

Considering histone modifications, we observed the expected profiles around promoters and across gene bodies (Barski et al., 2007) of mono-, di-, and trimethylated H3K4 (H3K4Me1, H3K4Me2, and H3K4Me3, respectively); acetyl-H3K9 (H3K9Ac); and acetyl-H3K27 (H3K27Ac) at active and repressed genes in both control and OG86-treated cells (Figures S1A–S1E). As expected, surrounding the promoters of upregulated genes, there was a strong, significant, and positive correlation of increased gene expression with increased H3K4Me3, H3K9Ac, and H3K27Ac ChIP signal in OG86-treated cells versus vehicle-treated cells. No significant correlation was observed, however, for the LSD1-demethylation targets H3K4Me1 or H3K4Me2 (Shi et al., 2004) (Figures 1G and S1F–S1H). At genes downregulated following OG86 treatment, while there was no significant reduction in H3K4Me3 or H3K9Ac ChIP signals (Figure S1F), there was a modest relative reduction of H3K4Me1, H3K4Me2, and H3K27Ac ChIP signals of uncertain significance (Figure S1H). With regard to global histone H3K4 methylation marks, as determined by western blotting, no difference was observed in THP1 cells cultured for 7 days in OG86 (Figure S1I). Similar ChIP-seq analyses of H3K9 modifications (Me1, Me2, and Me3) in control and OG86-treated cells were not informative and demonstrated no correlation between changes in gene expression and changes in co-localized histone marks (data not shown).

Using Model-based Analysis of ChIP-Seq, v.2 (MACS2), 18,937 LSD1 binding peaks met threshold criteria in DMSO-treated control THP1 AML cells. By a ratio of approximately 4.5:1, these were distributed over intronic and intergenic regions versus promoter regions (Figures 1H and 1I). To determine whether LSD1 inhibition led to the expected accumulation of H3K4Me1 and H3K4Me2 marks on chromatin surrounding LSD1 binding sites, we first focused our attention on the promoter regions of the 766 genes upregulated following LSD1 inhibition. We compared the changes in histone methylation and acetylation observed at LSD1-bound promoters (214/766, 28%) with those promoters lacking an LSD1 binding peak within 1 kb of the transcription start site (TSS) (552/766, 72%) and observed no significant differences (Figure 1J). We next identified 6,778 active intergenic enhancer regions (Table S3) (defined as regions at least 5 kb from an annotated gene exhibiting coincident H3K9Ac and H3K4Me2 peaks) and performed a similar analysis. 23% (1,556/6,778) exhibited a coincident LSD1 peak, and 77% (5,222/6,778) did not. Once more, no relative accumulation of ChIP signal for the LSD1 demethylation targets H3K4Me1 and H3K4Me2 marks was observed. Instead, at LSD1-bound enhancers, there was a modest decrease in signal for H3K4Me1 and a highly significant increase in signal for both H3K9Ac and H3K27Ac (Figure 1K).

These initial analyses demonstrate that the early and extensive transcriptional consequences of LSD1 inhibition by OG86 in THP1 AML cells are not immediately preceded by the selective accumulation of H3K4Me1 and H3K4Me2 (the targets of LSD1’s histone demethylase activity) at LSD1-bound active enhancers and upregulated promoters. Instead, selective accumulation at LSD1-bound active enhancers of H3K9Ac and K3K27Ac was observed.

### LSD1 Catalytic Activity Is Not Required for AML Cell Clonogenic Potential

**Figure 2:**
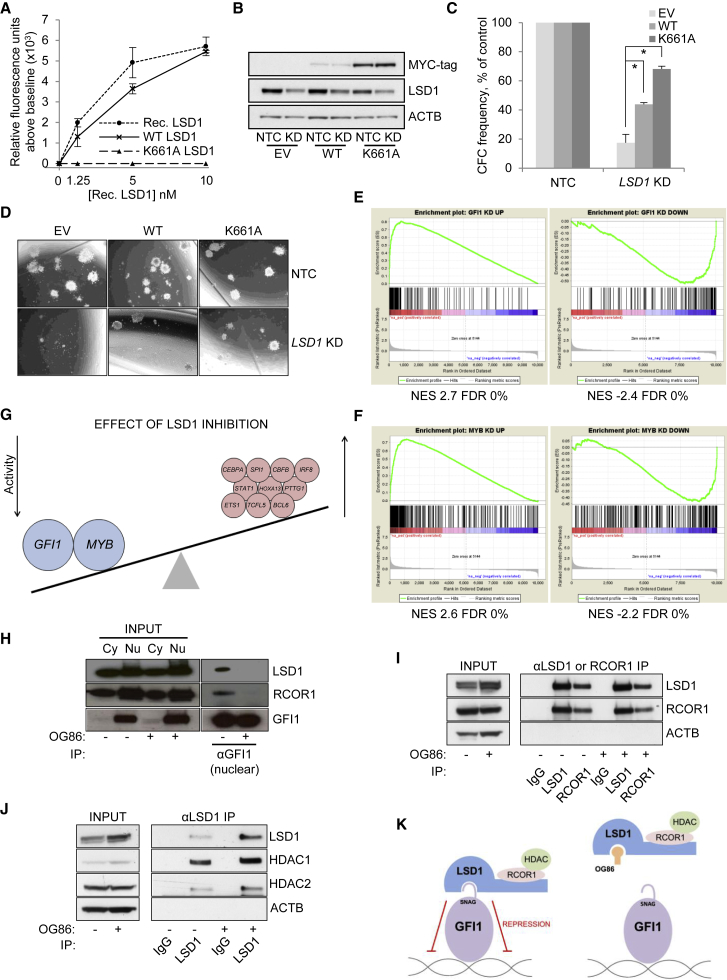
Catalytic Activity of LSD1 and Mimicry of LSD1 Inhibition by GFI1 Knockdown (A) Catalytic activity of recombinant LSD1, immunoprecipitated MYC-tagged wild-type (WT), or mutant (K661A) LSD1. Rec., recombinant. In (B)–(D), THP1 AML cells were infected with retroviruses expressing either WT or K661A mutant LSD1, or an empty vector (EV), with GFP as the selectable marker. FACS-purified GFP^+^ cells were then infected with lentiviruses expressing a shRNA-targeting *LSD1* for knockdown (KD) or a non-targeting control (NTC), with puromycin drug resistance as the selectable marker. (B) Western blot shows expression of the indicated proteins in the indicated conditions after 48 hr of drug selection. (C) Bar graph shows mean ± SEM for colony-forming cell (CFC) frequencies of drug-resistant cells relative to controls, enumerated after 10 days in semisolid culture (n = 3). ^∗^p < 0.05 for the indicated comparison using one-way ANOVA and Fisher’s least significant difference *post hoc* test. (D) Representative images of colonies from (C). (E and F) GSEA plots show enrichment of gene sets regulated by (E) *GFI1* KD or (F) *MYB* KD (Suzuki et al., 2009) among genes ranked according to fold change in expression following treatment of THP1 AML cells with 250 nM OG86 for 24 hr. (G) Image summarizes GSEA results. Blue circles indicate transcription factors where KD mimics transcriptional changes observed upon LSD1 inhibition. Pink circles indicate genes where KD induces downregulation of gene sets that are upregulated following LSD1 inhibition. Large circles indicate genes highlighted in (E) and (F). (H–J) THP1 AML cells were treated with 250 nM OG86 for 48 hr. Cell lysates were immunoprecipitated using (H) anti-GFI1, (I) anti-LSD1 or anti-RCOR1, and (J) anti-LSD1 in the indicated conditions, and western blots representative of at least three experiments are shown. IP, immunoprecipitation; Cy, cytoplasmic; Nu, nuclear. (K) Cartoon summarizes results of immunoprecipitation studies. See also Figure S2 and Tables S4 and S5.

To further investigate the requirement for the catalytic activity of LSD1 in the maintenance of AML cell clonogenic potential, we made use of an inactive K661A LSD1 mutant (Figures 2A and S2A) (Lee et al., 2006a, Adamo et al., 2011). By homology with maize polyamine oxidase, this conserved residue is hydrogen bonded to the N(5) atom of FAD via a water molecule and is essential to orientate FAD in the correct plane for flavin reduction during demethylation (Binda et al., 2001, Lee et al., 2005, Polticelli et al., 2005). *LSD1* knockdown (KD) using a lentiviral short hairpin RNA (shRNA) construct targeting the 3′ UTR substantially reduced the clonogenic potential of THP1 AML cells (Figures 2B–2D). Concomitant forced expression of wild-type (WT) *LSD1* partially rescued the KD phenotype (Figures 2B–2D). Of note, forced expression of K661A mutant LSD1 did likewise, with the greater degree of rescue likely due to a higher level of expression of the K661A versus the WT construct (Figure 2B). We performed similar experiments in murine MLL-AF9 AML cells with similar results. Forced expression of either human WT LSD1 or K661A mutant LSD1 in *Lsd1* KD cells (using a construct that does not target human *LSD1*) rescued clonogenic potential and differentiation block to an equivalent extent, as determined by analysis of colony morphology (Figures S2B–S2E). These data demonstrate that the catalytic activity of LSD1 is not required for the clonogenic potential of human THP1 or murine MLL-AF9 AML cells and further indicate that tranylcypromine-derivative pharmacologic inhibitors target histone demethylation-independent activities of LSD1.

### Pharmacologic Inhibition of LSD1 Mimics *GFI1* KD

Given the physical interaction of LSD1 with several transcription factors (Lynch et al., 2012), we next sought to determine whether its pharmacologic inhibition by OG86 mimics the transcriptional consequences of transcription factor KD. To address this, we identified gene sets with expression significantly up- or downregulated by at least 2-fold following siRNA-induced KD of 46 genes coding for transcription factors and other proteins. Transcriptome data were from a prior study that also made use of THP1 AML cells (Suzuki et al., 2009) (Table S4). Using gene set enrichment analysis (GSEA), we observed that only gene sets up- or downregulated by *GFI1* or *MYB* KD were concordantly enriched among those up- or downregulated following treatment of THP1 AML cells with OG86 (Figures 2E and 2F; Table S5). Thus, in THP1 AML cells, pharmacologic inhibition of LSD1 mimics depletion of *GFI1* or *MYB* transcripts (Figure 2G). *GFI1B* is not expressed in THP1 cells (Table S2).

Consistent with the increased expression of a myeloid differentiation program following OG86 treatment, among genes upregulated following LSD1 inhibition, there was also significant enrichment of gene sets whose expression is sustained by myeloid transcription factors such as SPI1 (PU.1), CEBPA, CBFB, and IRF8 (Figures 2G and S2F; Table S5).

### Pharmacologic Inhibition of LSD1 Impairs Interaction with GFI1 and Chromatin

Given that physical association of LSD1 with the N-terminal SNAG domain of GFI1 is essential for the function of GFI1 as a transcription repressor (Saleque et al., 2007), we evaluated whether OG86 disrupts this interaction. Indeed, in the absence of OG86, immunoprecipitation of endogenous GFI1 in THP1 AML cells readily pulled down endogenous LSD1, whereas in the presence of OG86, the interaction was disrupted (Figure 2H). A similar finding for GFI1B and LSD1 was recently reported with a related tranylcypromine derivative, T-3775440 (Ishikawa et al., 2017). In contrast, OG86 did not alter the interaction of LSD1 with CoREST complex members RCOR1, HDAC1, and HDAC2 (Figures 2I and 2J). Thus, pharmacologic inhibition of LSD1 disrupts its association with GFI1, potentially abrogating GFI1 activity (Figure 2K).

**Figure 3:**
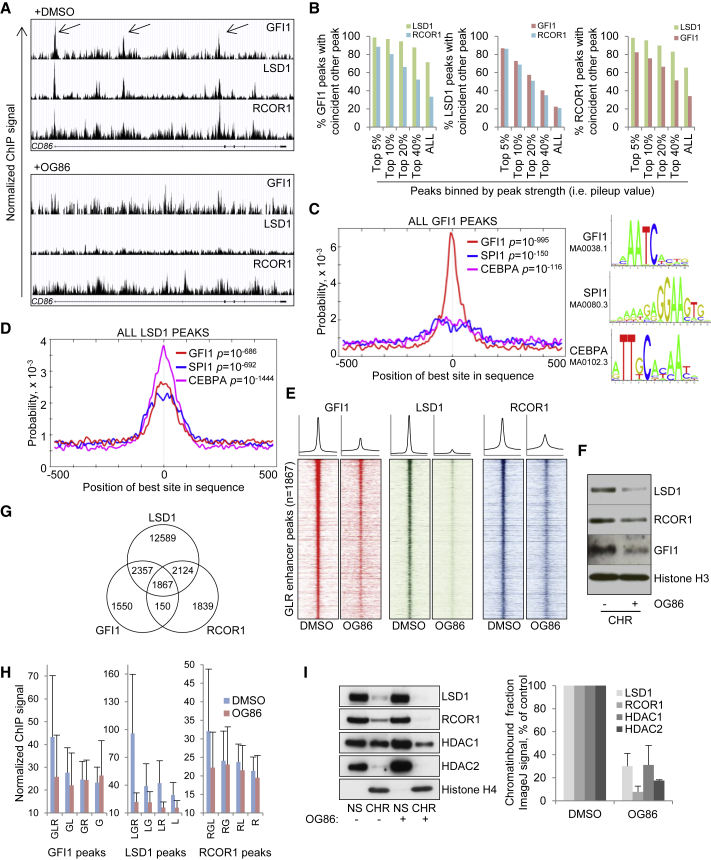
Close Physical Association of GFI1, LSD1, and RCOR1 on Chromatin and Its Abrogation by OG86 (A) Exemplar ChIP-seq tracks following treatment of THP1 AML cells with DMSO vehicle or 250 nM OG86 for 48 hr. Arrows indicate coincident GLR peaks. (B) Bar graphs show the percentage of GFI1 (left), LSD1 (middle), or RCOR1 (right) binding peaks with the indicated coincident binding peaks. (C and D) MEME-ChIP motif enrichment plots with indicative p values for (C) GFI1 peaks (n = 5,924) or (D) LSD1 peaks (n = 18,937). (E) Heatmaps show ChIP signal for the indicated proteins at 1,867 “GLR” peaks (peak apex ± 1 kb) ranked according to GFI1 peak strength. (F) Western blot shows the indicated chromatin-bound proteins in THP1 AML cells treated with DMSO vehicle or 250 nM OG86 for 48 hr. CHR, chromatin. (G) Venn diagram shows categories of binding peaks in THP1 AML cells. (H) Bar graph indicates means ± SD for ChIP signal at sites of the indicated proteins and the indicated peak categories in DMSO vehicle-treated cells and at the same sites in OG86-treated cells after 48 hr. G, GFI1; L, LSD1; R, RCOR1. (I) Murine MLL-AF9 AML cells were treated with 250 nM OG86 or DMSO vehicle for 48 hr followed by subcellular fractionation. Representative western blots (left) show the presence of the indicated proteins in the indicated cellular fractions in the presence or absence of OG86. The graph on the right shows means ± SEM for western blot signal in chromatin-bound fractions in the presence or absence of OG86, as determined by ImageJ densitometry (n = 3). NS, nuclear soluble; CHR, chromatin. See also Figure S3.

To explore the genome-wide association of GFI1 with LSD1 and RCOR1, we performed ChIP-seq using antibodies versus endogenous proteins in DMSO-treated control THP1 AML cells. Using MACS2, 5,924 GFI1 and 5,980 RCOR1 binding peaks met threshold criteria, and once more, peaks were predominantly distributed over intronic and intergenic regions; a greater proportion of RCOR1 peaks were promoter bound, likely in keeping with its contribution to protein complexes other than CoREST (Figures 1I, 3A, S3A, and S3B). While, overall, 71.3% of GFI1 peaks were coincident with an LSD1 peak (i.e., the GFI1 peak apex is within ±500 bp of an LSD1 peak apex), and 33.4% were coincident with an RCOR1 peak, nearly all of the strongest GFI1 peaks (based on MACS2 pileup value) exhibited coincident LSD1 and RCOR1 binding (i.e., 98.6% and 88.4% of the strongest 5% of GFI1 peaks were coincident with LSD1 and RCOR1 peaks, respectively) (Figures 3A, 3B, and S3C). A similar pattern was observed in the reverse analyses, with the strongest LSD1 and RCOR1 peaks being coincident with a GFI1 peak (i.e., 86.7% and 82.4% of the strongest 5% of LSD1 and RCOR1 peaks, respectively, were coincident with a GFI1 peak) (Figure 3B). These data demonstrate close physical association on chromatin of GFI1 with CoREST complex members LSD1 and RCOR1, with the strongest GFI1, LSD1, and RCOR1 binding peaks generally being coincident one with another.

MEME-ChIP (Machanick & Bailey, 2011) confirmed that genomic sequences at the center of GFI1 binding peaks were strongly enriched for the GFI1 consensus binding motif (Figure 3C). There was weaker but significant enrichment close to GFI1 binding peaks for CEBPA and SPI1 consensus binding motifs. These transcription factors have key roles in myeloid differentiation and are among the most highly expressed transcription regulator genes in THP1 AML cells (Table S2); their dependent gene sets were upregulated following treatment of THP1 cells with OG86 (Figure 2G; Table S5).

Motif enrichment analysis on the complete sets of LSD1 and RCOR1 binding peaks revealed, as expected, significant enrichment for GFI1 consensus motifs in genomic sequences surrounding peak centers (Figures 3D and S3D). However, in contrast to the pattern observed at GFI1 peaks, the central enrichment for CEBPA and SPI1 consensus motifs was relatively more pronounced, in keeping with LSD1 and RCOR1 binding to sites other than those bound by GFI1, albeit with significantly weaker peak strength. This was exemplified by motif enrichment analysis of two sets of LSD1 binding peaks: those co-localized with a GFI1 peak (LSD1^pos^GFI1^pos^ peaks; n = 4,172) and those not associated with a GFI1 peak (LSD1^pos^GFI1^neg^ peaks; n = 14,765). The LSD1^pos^GFI1^pos^ peaks bound genomic sequences strongly enriched for GFI1 motifs, whereas LSD1^pos^GFI1^neg^ peaks bound genomic sequences particularly enriched for CEBPA motifs (Figure S3E).

In keeping with the physical separation of LSD1 and RCOR1 from GFI1 induced by OG86 in immunoprecipitation experiments, we observed a loss of LSD1 and RCOR1 ChIP-seq signal in OG86-treated THP1 AML cells, indicating that drug treatment compromized the interaction of co-localized LSD1/RCOR1 with chromatin (Figures 3A, 3E, S3F, and S3G). Interestingly, we also observed a loss of GFI1 ChIP signal (Figures 3A and 3E), and a decrease in chromatin-bound GFI1 by western blot (Figure 3F), but unchanged levels of total nuclear GFI1 (Figure 2H), suggesting that physical interaction of LSD1/RCOR1 with GFI1 may be required to stabilize GFI1 on chromatin, as has been reported for the interaction of SNAI1 with LSD1 (Lin et al., 2010). Of note, the greatest proportional reduction in ChIP signal for each of GFI1, LSD1, and RCOR1 was at the 1,867 sites co-occupied by the three proteins (Figures 3E, 3G, 3H; Table S3), with lesser or absent proportional reduction at other categories of sites.

In OG86-treated cells, MACS2 analysis identified 3,102 LSD1 peaks, 536 GFI1 peaks, and 5,582 RCOR1 peaks (Figure S3H). The strongest GFI1, LSD1, and RCOR1 binding peaks in drug-treated cells were entirely or mostly a subset of the peaks observed in control cells, indicating that there was no significant redistribution of GFI1, LSD1, or RCOR1 binding sites (Figure S3I). The selective loss of LSD1 and RCOR1 from sites of GFI1 binding was further supported by motif analysis of residual LSD1 and RCOR1 binding peak sequences in OG86-treated cells. This revealed substantially reduced enrichment in particular for GFI1 consensus motifs (Figures 3D, S3D, S3J, and S3K).

To demonstrate OG86-induced loss of LSD1 from chromatin in an alternative species, we performed subcellular fractionation analyses in murine MLL-AF9 AML cells. Following drug treatment, LSD1 and its associated CoREST complex components shifted from the chromatin-bound fraction into the nuclear-soluble fraction (Figure 3I), as observed in THP1 AML cells (Figure 3F). Thus, treatment of AML cells with OG86 leads to physical separation of LSD1 from both the transcription factor GFI1 and chromatin.

Taken together, these analyses demonstrate that the strongest binding peaks for GFI1, LSD1, and RCOR1 in THP1 AML cells are coincident with one another and that pharmacologic inhibition of LSD1 in particular targets GFI1/CoREST chromatin-bound complexes for disruption and release to the nucleoplasm.

### OG86-Induced AML Cell Differentiation Depends upon Separation of LSD1 from GFI1

**Figure 4:**
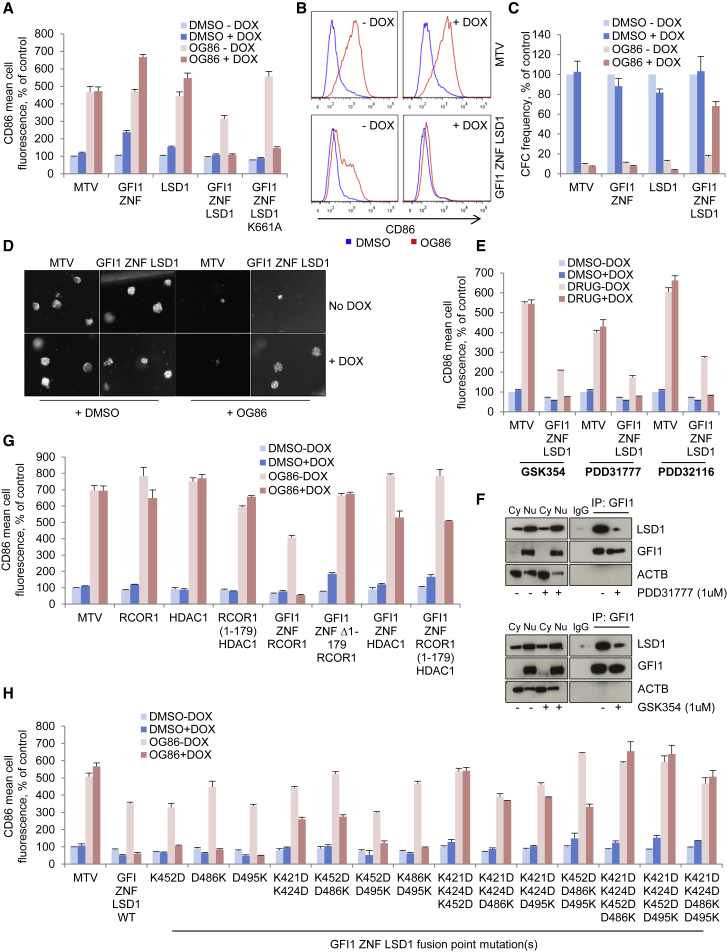
Displacement of LSD1/RCOR1 from GFI1 Is Required for OG86-Induced Myeloid Differentiation (A–H) THP1 AML cells infected with lentiviruses expressing GFI1 fusion or control constructs regulated by a doxycycline-regulated promoter were treated with 250 nM OG86 or DMSO vehicle in the presence or absence of doxycycline. Bar graphs in (A), (E), (G), and (H) indicate means ± SEM for CD86 mean cell fluorescence 24 hr later, as determined by flow cytometry, in the indicated conditions (n = 3 for each graph). (B) Representative flow cytometry histograms from (A). (C) Bar graphs indicate means ± SEM for colony-forming cell (CFC) frequency (n = 3 for each graph). Colonies were enumerated 10 days later. (D) Representative images from (C). (E) Same as in (A) but with 1 μM GSK354, 1 μM PDD31777, 5 μM PDD32116, or DMSO vehicle as indicated. (F) THP1 AML cells were treated with 250 nM OG86 for 48 hr. Cell lysates were immunoprecipitated using the indicated antibodies in the indicated conditions, and western blots representative of at least three experiments are shown. IP, immunoprecipitation; Cy, cytoplasmic; Nu, nuclear. See also Figure S4.

To provide functional evidence that the protein:protein interaction of LSD1 with GFI1 is the critical target of OG86 (rather than the demethylase activity of LSD1), we generated conditional constructs in which the DNA-binding domain of GFI1 was fused directly to LSD1 so that the two were no longer separable upon addition of LSD1 inhibitor (Figure S4A). This, in effect, renders GFI1 constitutively active (Saleque et al., 2007). Construct expression was induced in THP1 AML cells using a doxycycline-regulated system (Figure S4B). As expected, OG86 treatment of THP1 AML cells promoted differentiation, as evidenced by reduced clonogenic potential and increased expression of the monocyte/macrophage lineage differentiation marker CD86 (used as a surrogate for differentiation in the experiments that follow) (Figures 4A–4D). While either the zinc-finger DNA-binding domain of the transcription repressor GFI1 (GFI1 ZNF) or that of LSD1 modestly induced CD86 expression in both vehicle- and drug-treated cells, the GFI1 ZNF LSD1 fusion protein completely blocked upregulation of CD86 expression in response to OG86 treatment and rescued clonogenic potential (Figures 4A–4D). Similar experiments using a full-length GFI1-LSD1 fusion gave similar results (Figures S4C and S4D), as did experiments with a GFI1 ZNF LSD1 fusion with a K661A catalytic site mutation (Figure 4A). The reduction of CD86 expression in GFI1 ZNF LSD1 and GFI1 (full-length) LSD1 cells treated with OG86 in the absence of doxycycline (Figures 4A and S4C) was due to incomplete repression of the tetracycline response element (data not shown). These data confirm that OG86-induced myeloid differentiation in THP1 AML cells results from the physical separation of LSD1 from GFI1.

To determine whether this mechanism was generally applicable to other, structurally unrelated LSD1 inhibitors that also target the substrate interaction and catalytic site of LSD1, we repeated experiments using three different reversible inhibitors: (R)-4-(5-(pyrrolidin-3-ylmethoxy)-2-(p-tolyl)pyridin-3-yl) benzonitrile) (GSK354; Hitchin et al., 2013); 4-[3-[(3R)-3-aminopiperidine-1-carbonyl]-5-[(3-ethylisoxazol-5-yl)methoxy]pyrazol-1-yl] benzonitrile (Compound 11p; PDD31777; Mould et al., 2017a); and 4-[[2-(2,7-Diazaspiro[3.5]nonan-7-yl)-2-oxo-ethyl]-[(3-fluoro-4-methoxy-phenyl)methyl]amino] benzonitrile (Compound 32; PDD32116; Mould et al., 2017b) (Figure S4E). In each case, we found that drug-induced upregulation of the differentiation-linked cell-surface marker CD86 could be prevented by expression of the GFI1 ZNF-LSD1 fusion (Figure 4E) and that the interaction of GFI with LSD1 was impaired (Figure 4F; data not shown). This confirms that LSD1 inhibitors, in general, induce displacement of LSD1 from its interaction with GFI1 to promote myeloid differentiation.

Our experimental system afforded us the opportunity to further explore molecular interactions capable of rendering GFI1 constitutively active and, thus, able to block the differentiation-promoting activity of LSD1 inhibition. We first generated a conditional GFI1 ZNF RCOR1 expression construct and observed that, while induced expression of RCOR1 alone had no effect, expression of GFI1 ZNF RCOR1 also rendered THP1 AML cells resistant to OG86 (Figure 4G). We next generated a series of conditional GFI1 ZNF LSD1 fusion constructs with point mutations in the LSD1 component of the protein, which were predicted to disrupt, through altered amino acid polarity, the interaction of the Tower domain of LSD1 with RCOR1 (Figure S4F). These mutants were used to confirm and evaluate the significance of the interaction between LSD1 and RCOR1 in acting as effectors of the activity of GFI1 as a transcription repressor. While K452D, D486K, and D495K single mutations (in the LSD1 sequence) had little or no adverse effect, two double-mutant constructs (KK421/424DD and K452D/D486K) partially impaired fusion activity, while three triple mutants (KK421/424DD+K452D, KK421/424DD+D486K, and KK421/424DD+D495K) fully impaired the ability of the fusion to block upregulation of the differentiation marker CD86 (Figures 4H and S4F). This was, likewise, the case with the three quadruple mutant constructs tested (Figure 4H) and suggests that mutations targeting more than one point of interaction between RCOR1 and LSD1 on the Tower domain are required to fully block the interaction of the two proteins (Figure S4F). Immunoprecipitation experiments using one of the quadruple GFI1 ZNF LSD1 fusion mutants confirmed its inability to interact with RCOR1 (Figure S4G). These data, again, demonstrate the critical role of displacement of LSD1/RCOR1 from GFI1 in OG86-induced myeloid differentiation but, critically, also show that the activity of GFI1 as a transcription repressor depends upon the recruitment of RCOR1 by the Tower domain of LSD1 to sites of GFI1 binding.

To further explore the mechanism of GFI1-mediated transcription repression, we generated a conditional GFI1 ZNF RCOR1 expression construct that lacks the first 179 amino acids of RCOR1 (GFI1 ZNF Δ1–179 RCOR1). This N-terminal portion of RCOR1 contains an HDAC1/2 recruitment domain that has been implicated in the co-repressor activity of RCOR1 (You et al., 2001). In contrast to the full-length GFI1 ZNF RCOR1 fusion, expression of GFI1 ZNF Δ1–179 RCOR1 entirely failed to prevent drug-induced upregulation of CD86 (Figure 4G). This experiment, and those described earlier, suggested that critical transcription repressors are recruited through the N-terminal portion of RCOR1 via LSD1 to GFI1 binding sites on chromatin. Indeed, conditional expression of either a direct GFI1 ZNF HDAC1 fusion or a three-way fusion involving the GFI1 DNA binding domain and the N-terminal 179 amino acids of RCOR1 and HDAC1—GFI1 ZNF RCOR1 (1–179) HDAC1—conferred highly significant resistance of cells to OG86, although not as substantial as that achieved with the GFI ZNF LSD1 and GFI ZNF RCOR1 fusions (Figure 4G).

Together, these data demonstrate that, in THP1 AML cells, both irreversible and reversible pharmacologic inhibitors of LSD1 displace the LSD1/RCOR1 complex from its physical interaction with GFI1 and that loss of histone deacetylase activity at GFI1 binding sites is, at least in part, responsible for differentiation.

### OG86-Induced Eviction of LSD1 from Chromatin Increases Activity of GLR-Bound Enhancers

We next evaluated changes in histone modifications and chromatin accessibility (using the assay for transposase-accessible chromatin sequencing; ATAC-seq) surrounding sites co-occupied by GFI1, LSD1, and RCOR1 (Figure 3G), 24 hr following LSD1 inhibition with OG86. To facilitate these analyses, and to provide a comparator for any observed changes at GFI1 binding sites, we also performed ChIP-seq for the transcription activator MYB. Using MACS2, we identified 47,818 MYB binding peaks in DMSO-treated control THP1 AML cells, which were again predominantly distributed over intronic and intergenic versus promoter regions. MYB binding peak profiles and distributions did not change substantially following treatment of cells with OG86 (Figures S5A–S5C). Analysis of genomic sequences at binding peaks using MEME-ChIP confirmed strong enrichment for MYB consensus binding motifs (Figure S5D).

**Figure 5:**
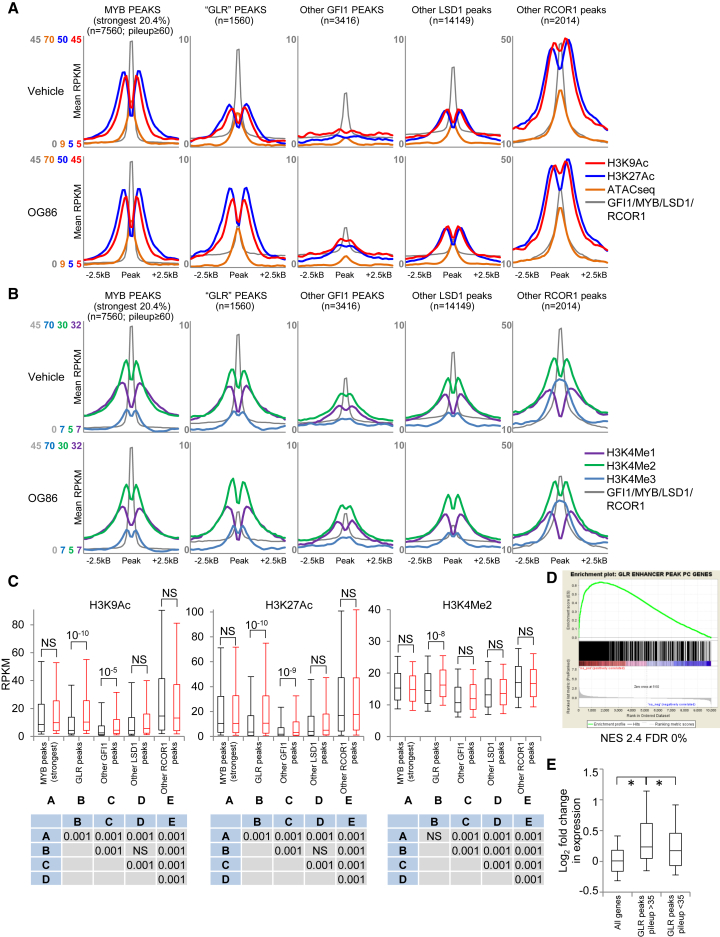
Chromatin Profiles Surrounding Transcription Factor Binding Sites THP1 AML cells were treated with 250 nM OG86 or DMSO vehicle for 24 hr. (A and B) Graphs indicate (A) mean ChIP-seq signal for H3K9Ac and H3K27Ac and ATAC-seq signal or (B) mean ChIP-seq signal for H3K4Me1, H3K4Me2, and H2K4Me3 (reads per kilobase per million mapped reads; RPKM) surrounding the indicated MYB, GFI1/LSD1/RCOR1 coincident peaks, “GLR” peaks, or other GFI1, other LSD1, or other RCOR1 peaks at intronic and intergenic binding sites (i.e., putative enhancers) in the indicated conditions. (C) Box-and-whisker plots show median, 25^th^, and 75^th^ percentile values (box) and 10^th^ and 90^th^ percentile values (whiskers) for normalized ChIP signal for H3K9Ac, H3K27Ac, and H3K4Me2 surrounding (± 1 kb) the indicated peak categories. Black boxes indicate DMSO vehicle; red boxes indicate OG86. The p values (t test) are shown for the indicated comparisons. NS, not significant. Tables beneath box-and-whisker plots show p values for comparisons of signal at the indicated peak categories (labeled A–E) in the DMSO vehicle condition, as determined by one-way ANOVA and Tukey’s honest significant difference *post hoc* test. (D) GSEA plot. NES, normalized enrichment score; FDR, false discovery rate. (E) Box-and-whisker plots show median, 25^th^, and 75^th^ percentile values (box), and 10^th^ and 90^th^ percentile values (whiskers) for log_2_ fold change in expression of all expressed protein coding genes or those located next to stronger (pileup ≥ 35) or weaker (pileup < 35) “GLR” peaks. ^∗^p < 0.001, as determined by one-way ANOVA and Tukey’s honest significant difference *post hoc* test. See also Figure S5.

We confined our analysis to chromatin surrounding GFI1, LSD1, RCOR1, and MYB binding peaks found in intronic and intergenic regions (rather than promoter regions) to focus on putative enhancers; the distribution of histone modifications is quite distinct at promoters versus enhancers. We identified 1,560 sites where above-threshold ChIP peaks for GFI1, LSD1, and RCOR1 coincided (hereinafter “GLR” peaks). Read distribution profiles surrounding transcription factor binding sites were as expected (Figures 5A–5C and S5E–S5G). By comparison with chromatin surrounding the strongest MYB binding peaks, there was a significantly lower signal for H3K9Ac, H3K27Ac, and chromatin accessibility at GLR peaks in vehicle-treated cells, whereas the ChIP signal for H3K4Me1, H3K4Me2, and H3K4Me3 was no different. ChIP and ATAC-seq profiles surrounding “other GFI1” peaks (n = 3,416), “other LSD1” peaks (n = 14,149), and “other RCOR1” peaks (n = 2,014) were consistent with occupancy of enhancer sites by these proteins with, on average, increasing activity respectively (Figures 5A–5E and S5E–S5G).

Following pharmacologic inhibition of LSD1 with OG86, the most substantial and significant changes in histone modifications were observed for acetylation surrounding GLR peaks. While there was no significant change in histone acetylation surrounding the strongest MYB sites, or at “other LSD1” or “other RCOR1” sites, at GLR sites, the mean ChIP-seq signal for H3K9Ac and H3K27Ac increased by 48% and 63%, respectively (Figures 5A–5C). There was also a much more modest but, nevertheless, significant increase in histone acetylation surrounding the set of “other GFI1” peaks (Figures 5A–5C). Division of this group into GFI1^pos^LSD1^pos^RCOR1^neg^ and GFI1^pos^LSD1^neg^RCOR1^neg^ peaks revealed that significantly increased histone acetylation was associated exclusively with the former set (Figures S5H and S5I). One possible explanation is that the RCOR1 ChIP efficiency was inferior to that of the LSD1 ChIP and that the GFI1^pos^LSD1^pos^RCOR1^neg^ sites, in fact, exhibit biologically relevant RCOR1 binding, which was sub-threshold in this analysis.

The link between the changes in histone acetylation surrounding GLR peaks and increased transcription of nearby genes was confirmed by GSEA. The 1,560 GLR enhancer peaks mapped to 1,334 protein coding genes (Table S7). Remarkably, the most strongly enriched biological process terms in Gene Ontology analysis of this gene set were “GO:0000122∼negative regulation of transcription from RNA polymerase II promoter” and “GO:0045893∼positive regulation of transcription, DNA-templated” (Table S7), in keeping with the role of GFI1 in controlling expression of a multitude of transcription factor genes in myeloid cells, including those such as *IRF8*, *KLF4*, and *MEF2C* with roles in monocyte/macrophage differentiation. There was a highly significant enrichment of this gene set (Table S7) among upregulated genes (Figure 5D). Genes close to stronger GLR peaks (GFI1 pileup value, >35) exhibited significantly higher fold change increases in expression, in comparison with those close to weaker GLR peaks (GFI1 pileup value, <35) (Figure 5E), indicating that greater peak strength was linked to greater transcription repression.

**Figure 6:**
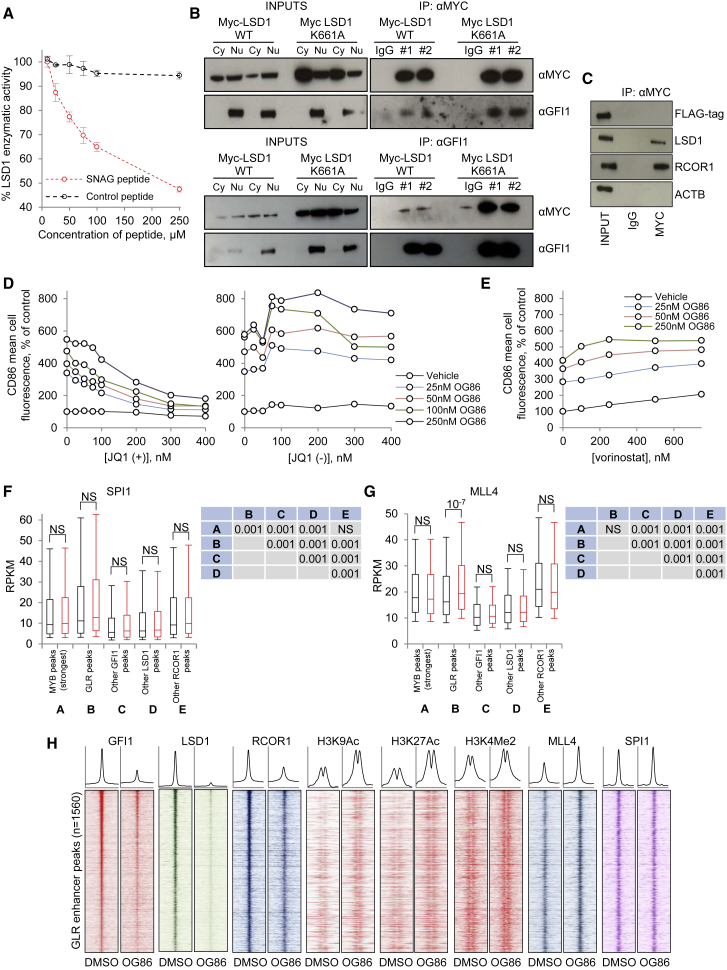
LSD1 Inhibition Causes GFI1-Bound Enhancer Activation (A) Catalytic activity of recombinant LSD1 versus H3K4Me1 in the presence of a GFI1 SNAG domain or control peptide. (B and C) THP1 AML cells were infected with retroviruses expressing MYC- or FLAG-tagged WT or MYC-tagged K661A mutant LSD1 and selected with puromycin. Cell lysates were immunoprecipitated using (B) anti-MYC tag or anti-GFI1, or (C) anti-MYC tag, in the indicated conditions, and western blots representative of two experiments (#1 and #2) are shown. IgG, immunoglobulin G; IP, immunoprecipitation; Cy, cytoplasmic; Nu, nuclear. (D and E) Graphs shows dose-response curves for THP1 cells treated with OG86 and (D) JQ1 (+ and − enantiomers) and (E) vorinostat. (F and G) Box-and-whisker plots show median, 25^th^, and 75^th^ percentile values (box), and 10^th^ and 90^th^ percentile values (whiskers) for normalized ChIP signal for (F) SPI1 and (G) MLL4 surrounding (± 1 kb) the indicated peak categories. Black boxes indicate DMSO vehicle; red boxes indicate OG86. The p values (t test) are shown for the indicated comparisons. NS, not significant. Tables at the side of box-and-whisker plots show p values for comparisons of signal at the indicated peak categories (labeled A–E) in the DMSO vehicle condition, as determined by one way ANOVA and Tukey’s honest significant difference *post hoc* test. (H) Heatmaps show ChIP signal for the indicated proteins at 1,560 “GLR” putative enhancer binding peaks (peak apex ± 1 kb), ranked according to GFI1 peak strength. See also Figure S6.

Considering histone methylation and chromatin accessibility, the only significant change observed was a modest (mean 9%) increase in H3K4Me2 ChIP signal on chromatin surrounding GLR peaks (Figures 5A–5C and S5E–S5G). Specifically, 24 hr following the addition of OG86, there was no significant accumulation of H3K4Me2 ChIP signal surrounding “other LSD1” peaks or accumulation of H3K4Me1 ChIP signal surrounding either GLR peaks or “other LSD1” peaks as would be expected if LSD1 were constitutively demethylating histone tails at its bound locations. This was the case whether all peaks were considered or only a subset of the strongest peaks by pileup value was considered (Figures S5J and S5K). Multiple lines of evidence demonstrate that the modest increase of H3K4Me2 ChIP signal at GLR peaks 24 hr following OG86 treatment of cells was not due to inhibition of the demethylase activity of LSD1 at these sites. First, LSD1 is unable to demethylate histone tails while the SNAG domain of GFI1, which mimics the structure of the N-terminal tail of histone H3 (Baron et al., 2011), occupies its substrate-binding pocket. In keeping with this, a GFI1 SNAG domain peptide dose dependently inhibited the demethylase activity of LSD1 versus a histone H3 (1–21) K4 mono-methylated peptide (Figure 6A). Second, the demethylase activity of LSD1 was not required to sustain the clonogenic potential of AML cells (Figures 2 and S2A–S2E), and a GFI1 ZNF LSD1 K661A demethylase mutant was as effective as the WT fusion in preventing OG86-induced upregulation of differentiation markers (Figure 4A). Related to this, and as expected, LSD1 K661A interacts with GFI1 with an efficiency equivalent to that of WT LSD1 (Figure 6B), demonstrating that the demethylase dead version of the enzyme is fully able to provide the normal structural functions of LSD1 to recruit other COREST complex components. Third, in immunoprecipitation experiments, while the interaction of MYC-tagged LSD1 with endogenous RCOR1 is readily observed, there was no interaction with a co-expressed FLAG-tagged version of LSD1, demonstrating that LSD1/RCOR1 does not recruit additional LSD1 to sites of GFI1 interaction through dimerization (Figure 6C). Furthermore, in a time course experiment (Figure S6A), while substantial increases in both transcription and enhancer H3K27Ac acetylation were observed within 2 hr of OG86 treatment, no changes in H3K4Me2 were observed.

To provide further evidence for the role of increased acetylation at GFI1/LSD1-bound enhancers as a critical mediator of drug-induced myeloid differentiation, we co-treated THP1 AML cells with OG86 and the active enantiomer of the BRD2/3/4 bromodomain inhibitor JQ1. Bromodomain-containing proteins are key epigenetic readers of histone acetylation marks. We observed a dose-dependent inhibition of CD86 upregulation with JQ1, but not with the inactive enantiomer JQ1(−) (Figure 6D). We also observed a modest additive and dose-dependent increase in CD86 expression with vorinostat, a class 1 and class 2 histone deacetylase inhibitor (Figure 6E).

We next investigated whether, 24 hr following OG86 treatment, the modest increase in histone H3K4 dimethylation at GLR occupied sites (which, together with higher acetylation, is in keeping with the increased activation state of enhancers) might be explained by the recruitment of a histone methyltransferase following drug treatment. To address this question, we performed ChIP-seq for the H3K4 mono- and dimethyltransferase MLL4. To provide further insight into the function of GLR bound enhancers and their mechanism of increased activation following OG86 treatment of AML cells, we also performed ChIP-seq for the myeloid master regulator transcription factor SPI1 (PU.1), whose gene set is upregulated following OG86 treatment of AML cells.

Using MACS2, 102,807 PU.1 and 23,382 MLL4 binding peaks met threshold criteria, and peaks were predominantly distributed over intronic and intergenic regions; a greater proportion of MLL4 peaks was promoter associated. MEME-ChIP confirmed strong enrichment for SPI1 motifs at the center of SPI1 binding peaks and strong enrichment for SPI1 and CEBPA motifs at the center of MLL4 binding peaks (Figures S6B–S6C). Interestingly, there was a significantly higher ChIP signal for SPI1 at GLR peaks in comparison with MYB peaks and other LSD1, GFI1, and RCOR1 peaks (Figure 6F), but following OG86 treatment of THP1 AML cells, no change was observed. By contrast, the only significant difference in ChIP signal between control and OG86-treated cells for MLL4 was observed at GLR peaks, where there was a mean 16% increase (Figures 6G, 6H, and S6D).

**Figure 7:**
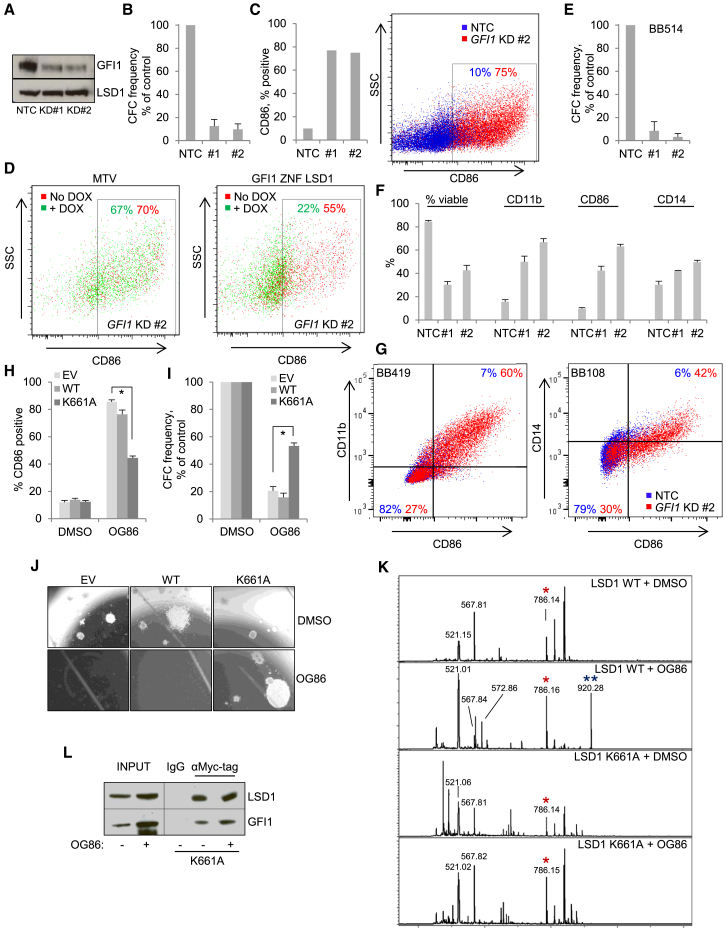
GFI1 Targeting in Primary AML Cells and a Candidate Resistance Mechanism AML cells were infected with lentiviruses targeting *GFI1* for KD or a non-targeting control (NTC) with puromycin drug resistance as the selectable marker. (A) Western blot shows GFI1 KD in THP1 AML cells. (B–G) In (B) and (E), bar graphs show means ± SEM for colony-forming cell (CFC) frequencies of drug-resistant (B) THP1 AML cells or (E) primary patient MLL-AF9 AML cells relative to controls, enumerated after 10 days in semisolid culture (n = 3). In (C) and (F), graphs and FACS plots show upregulation of the indicated myeloid maturation markers 96 hr following *GFI1* KD in (C) THP1 cells or (F) primary patient MLL-translocated AML cells (n = 5 separate patients). (D) *GFI1* KD was initiated in THP1 AML cells with conditional expression under doxycycline control of GFI1 ZNF LSD1 or an empty vector control (MTV). FACS plots show the percentage of CD86-positive cells 96 hr following initiation of *GFI1* KD and treatment of cells with doxycycline or vehicle. (G) Exemplar FACS plots from (F). (H–L)THP1 AML cells were infected with retroviruses expressing MYC-tagged WT or K661A mutant LSD1, or an empty vector (EV), with GFP as the selectable marker. GFP^+^ cells were treated with 250 nM OG86 or DMSO vehicle control. (H and I) Bar graphs show (H) means ± SEM for CD86 cell-surface expression for the indicated lines 24 hr following drug treatment, as determined by flow cytometry (n = 3) or (I) means ± SEM for colony-forming cell (CFC) frequencies relative to control cells for the indicated lines enumerated after 10 days in semisolid culture (n = 3). Asterisks indicate p < 0.001 for the indicated comparisons, as determined by one-way ANOVA followed by Fisher’s least significant difference *post hoc* test. (J) Representative images from (H). (K) Recombinant WT or K661A mutant LSD1 was incubated in the presence or absence of 250 nM OG86 for 2 hr at 25°C and then subjected to MALDI-TOF mass spectrometry. Red asterisks on the spectra indicate ions of the correct mass for FAD. Two blue asterisks indicate an ion of the correct mass for an FAD-tranylcypromine conjugate. (L) Forty-eight hours following drug treatment, cells were lysed, and lysates were subjected to anti-MYC tag immunoprecipitation. Representative western blot shows immunoprecipitation of GFI1 by a MYC-tagged LSD1 K661A mutant in the indicated conditions. See also Figure S7.

To provide further evidence that GFI1’s activity is the predominant target of LSD1 pharmacologic inhibition in AML cells, we performed genetic KD of *GFI1* in THP1 AML cells and observed a loss of clonogenic potential with the upregulation of CD86 with two separate KD constructs (Figures 7A–7C). The KD phenotype was an on-target consequence of GFI1 depletion, because upregulation of CD86 was abrogated by induced expression of the GFI1 ZNF LSD1 fusion but not the empty vector control (Figure 7D). Similar experiments using primary patient MLL-translocated AML cells from five separate patients yielded similar results, with upregulation of multiple myeloid-lineage markers, increased apoptosis, and reduced clonogenic potential (Figures 7E–7G; Table S1).

All together, these data demonstrate that LSD1 inhibition causes separation of LSD1/CoREST from GFI1 at SPI1-bound enhancers, with the most important consequence being localized increases in histone acetylation and consequent increased transcription of nearby genes.

### Binding of OG86 to FAD Requires Lysine 661 of LSD1, and Mutation of This Residue Renders Cells Drug Resistant

Finally, we examined a potential mechanism of drug resistance. Given that both WT LSD1 and the K661A catalytic mutant are able to rescue the clonogenic potential of *LSD1* KD THP1 AML cells, we tested the effects of OG86 treatment on cells expressing these constructs. The expected induction of CD86 and loss of clonogenic potential was observed in control cells and those expressing WT LSD1, but cells expressing the K661A catalytic mutant exhibited resistance to OG86 (Figures 7H–7J). Similar results were observed in experiments using murine MLL-AF9 cells (Figures S7A–S7C). These data were explained by an inability of OG86 to bind FAD in the presence of the K661A LSD1 mutation. In MALDI-TOF analyses (Figure 7K), while we observed peaks at 786 Da and 920 Da in OG86-treated WT LSD1 (corresponding to FAD and a FAD-tranylcypromine adduct; Schmidt and McCafferty, 2007), only the 786-Da FAD peak was observed in OG86-treated K661A mutant LSD1. An additional peak at 573 Da was also only seen in the OG86-treated WT LSD1 condition and corresponds to an adduct of flavin mononucleotide and tranylcypromine (Schmidt and McCafferty, 2007). It remains unclear whether OG86 is hydrolyzed upon initial binding to FAD or during MALDI-TOF.

As the K661A LSD1 catalytic mutant is unable to bind OG86, we investigated whether OG86-induced physical separation of LSD1 and GFI1 occurs in the presence of the mutant enzyme. THP1 AML cells expressing MYC-tagged versions of WT and K661A LSD1 were treated with OG86 or DMSO vehicle, and lysates were immunoprecipitated with an anti-GFI1 antibody. While OG86 treatment leads to loss of interaction of GFI1 with WT LSD1 (Figure 2H), and the K661A mutant interacts with GFI1 with an efficiency similar to that of WT LSD1 (Figure 6B), in the presence of OG86, the interaction of GFI1 with the K661A mutant was sustained (Figure 7L). Taken together, these data demonstrate that, in the same way that K661 facilitates the correct orientation of FAD for the normal demethylation reaction (Binda et al., 2001, Lee et al., 2005, Polticelli et al., 2005), it is also likely required to correctly orient FAD for covalent binding to OG86.

## Discussion

Early-phase clinical trial data indicate that the tranylcypromine-derivative LSD1 inhibitor ORY-1001 induces morphologic blast cell differentiation and differentiation syndromes in patients with *MLL*-translocated AML (Somervaille et al., 2016). Using a tractable experimental system and confirmatory analyses in patient cells, we now show that both irreversible and reversible inhibitors of LSD1 promote differentiation through disruption of the protein:protein interaction of GFI1 with LSD1 rather than impairment of histone demethylation. Our studies reveal a critical role for both GFI1 and LSD1 as key contributors to the cardinal pathologic feature of *MLL*-translocated AML, the differentiation block of immature blast cells.

Given the well-established ability of LSD1 to remove monomethyl and dimethyl marks from H3K4, we expected to see an accumulation of these modifications at LSD1 binding sites genome-wide following LSD1 inhibition and that this would precede and drive localized increases in transcription. Instead, 24 hr following drug treatment, at upregulated promoters or at active intergenic enhancers, the presence of an LSD1 binding peak made no difference to the change in H3K4 methylation status. Furthermore, the cellular consequences of LSD1 transcript depletion could be rescued equally well by a K661A LSD1 mutant (which retains structural but not catalytic activity) as by expression of the WT protein. These data, together with our finding of the importance of the role of the protein:protein interaction of LSD1 with GFI1, demonstrate that, in biology, LSD1 has distinct functions, both catalytic and structural.

The catalytic activity of LSD1 is well conserved through evolution and is likely essential for key aspects of mammalian biology. Indeed, in embryonic stem cell culture systems, the demethylase activity of LSD1 is required to maintain cells in an undifferentiated state (Adamo et al., 2011) and to maintain repression of *Pou5f1* (*Oct4*) (Nair et al., 2012). However, LSD1 also directly interacts with SNAG-domain family transcription repressors such as SNAI1, SCRT1, GFI1, and GFI1B through its substrate binding and recognition cleft, with the SNAG amino acid sequence as a molecular mimic for the histone H3 tail (Baron et al., 2011). When the binding cleft is occupied by a SNAG domain, there is no access for histone tails to the catalytic activity of LSD1, as we have confirmed using a synthetic GFI1-SNAG peptide. Indeed, in immunoprecipitation experiments with SNAI1 and LSD1, a SNAG peptide (1–17) was much more effective at blocking the interaction of the two proteins than histone tail peptides H3K4Me0 and H3K4Me2 (1–21), suggesting that the affinity of LSD1 for SNAG domain transcription factors is as strong as, if not stronger than, that for histone tails (Lin et al., 2010). The interaction of LSD1 with the SNAG-domain transcription factor GFI1 is essential for its function as a transcription repressor, because a P2A SNAG domain point mutation blocks the ability of LSD1 to bind GFI1 and also inactivates GFI1 as a transcription repressor (Grimes et al., 1996, Saleque et al., 2007). Of note is that, while, in our experiments, we found substantial coincidence of the strongest LSD1, RCOR1, and GFI1 binding peaks genome-wide, in embryonic stem cells, LSD1 and RCOR1 exhibited minimal overlap (Whyte et al., 2012), demonstrating that, in different cell types, LSD1 exhibits different interactions with chromatin through distinct complexes.

While accumulation of H3K4Me1/2 marks has been reported in AML cells following LSD1 inhibition (Schenk et al., 2012, McGrath et al., 2016), it is not clear that this is a direct and localized consequence of the blockade of the catalytic activity of LSD1 rather than an indirect consequence of enhancer activation at sites distant from LSD1 binding. For example, the KDM5 family of Jumonji domain demethylases also demethylate H3K4, and the duration of the analyses may give ample time for multiple indirect effects of LSD1 inhibition to become manifest.

Our investigations indicate that, in THP1 AML cells, the strongest LSD1 binding peaks are co-localized with the strongest GFI1 and RCOR1 binding peaks on chromatin and that, following drug treatment, LSD1 is displaced from these GLR sites. It is, therefore, unsurprising that the transcriptional and functional studies we performed support the LSD1:GFI1 interaction being of paramount importance as the target of LSD1 inhibitor activity. The marked increase in histone acetylation surrounding GLR binding sites, but not at the numerically greater “other LSD1” binding sites, indicates that a critical role of LSD1 is to serve as a platform for recruitment to GFI1 binding sites of histone deacetylase activity, most likely provided by HDAC1 and HDAC2 (You et al., 2001), and possibly also to block access of histone acetyltransferases. The marked increase in histone acetylation surrounding GLR binding sites is consistent with increased activation of this SPI1-bound enhancer set, which, interestingly, occurred without significant increase in chromatin accessibility over the 24-hr time course. The presence of SPI1 at GLR enhancers may provide an explanation for the observation that murine leukemias with reduced PU.1 levels are relatively resistant to the consequences of LSD1 inhibition (Cusan et al., 2018). While the importance of the role of HDAC recruitment to SNAG-domain transcription factor binding sites has been hypothesized (Chiang and Ayyanathan, 2013), our studies reveal the rapid and dynamic nature of the changes in histone acetylation following the displacement of LSD1/CoREST from GFI1 and chromatin, which take place within hours. While LSD1 was also lost from “other LSD1” binding sites, it remains unclear how LSD1 associates with chromatin at these more active enhancer sites and what its functional role might be, although, given the particular enrichment of its consensus motifs, we hypothesize that LSD1, as part of the NuRD complex (Whyte et al., 2012), may interact with CEBPA.

The central role of increased histone acetylation surrounding GFI1 binding sites as the key downstream consequence of pharmacologic inhibition of LSD1, as well as its direct link with transcription, is emphasized by several observations. First, it is well appreciated that the turnover rate of histone modifications is variable. In keeping with our observations, histone acetylation marks may turnover in minutes to hours, whereas turnover of histone methylation occurs over a much longer timescale. For example, the half-life of H3K4Me1 is 19 hr, and that for H3K27Me3 is 72 hr (Barth and Imhof, 2010). The rapid increases in transcription that we observe are in keeping with regulation by a histone modification with rapid rather than slow turnover. Second, the upregulation of the key differentiation marker CD86 was blocked by use of the bromodomain inhibitor JQ1, which prevents recognition of histone acetylation marks by BRD4 bound to the positive transcription elongation factor (pTEFb) complex. Third, the use of a non-selective histone deacetylase inhibitor induced expression of CD86 in its own right. While there was a modest (9%) increase in signal for H3K4Me2 (but not H3K4Me1) specifically at GLR sites 24 hr after drug treatment, this is in keeping with increased enhancer activation and likely explained by binding of the histone H3K4 dimethyltransferase MLL4. It is not the driver for the observed changes in transcription for the aforementioned reasons, the ability of the K661A mutant to rescue *LSD1* KD, and the ability of the GFI1 ZNF LSD1 K661A mutant fusion to rescue the effect of OG86 on promoting upregulation of CD86.

The ability of the GFI1 ZNF HDAC1 fusion to partially mimic constitutively active GFI1 and, thus, to prevent drug-induced differentiation further emphasizes the significance of acetylation at GFI/LSD1/RCOR1-bound enhancers in regulating transcription of nearby genes. We speculate that the reason why the GFI1 ZNF LSD1 and GFI1 ZNF RCOR1 fusions were more effective in mimicking constitutively active GFI1, and preventing OG86-induced differentiation, perhaps relates to the ability of these fusions to assemble on chromatin a properly constituted repressor complex of the correct stoichiometry and orientation. Such a complex may also serve to physically obstruct access of transcriptional activators such as EP300 and MLL4, in addition to recruiting HDAC activity.

Further supporting the concept that LSD1 inhibitors induce phenotypic consequences in hematopoiesis through the SNAG domain displacement mechanism, it is interesting to note that adult mice treated with inhibitors of LSD1 (Harris et al., 2012), or in whom LSD1 is depleted (Sprüssel et al., 2012) or deleted (Kerenyi et al., 2013), exhibit anemia, thrombocytopenia, and neutropenia but enhanced monocytopoiesis. These phenotypes bear a remarkable similarity to an aggregate phenotype for *Gfi1*^−/−^ mice (which exhibit severe neutropenia, a monocytosis, and normal numbers of red cells and platelets; Karsunky et al., 2002) and *Gfi1b*^−/−^ mice (which die *in utero* with arrested erythroid and megakaryocytic development; Saleque et al., 2002). The phenotypic effects of transcription factor knockout are in keeping with expression patterns for *Gfi1* versus *Gfi1b*: the former is expressed in myeloid lineages, whereas the latter is expressed in erythroid and megakaryocytic lineages (Saleque et al., 2002).

While it is interesting that drug discovery programs focusing on inhibition of demethylase activity as a target have generated compounds that function through an unexpected mechanism, the possibility is raised that a search for compounds that maximally disrupt SNAG domain:LSD1 interactions might yield molecules with higher potential therapeutic efficacy. The precise mechanism by which the protein:protein interaction is impaired remains unclear. Tranylcypromine and its derivatives when bound to FAD are too small to obstruct SNAG domain or histone tail access to the binding site cleft and may, therefore, function through an allosteric mechanism. In fact, molecular dynamic modeling studies suggest that LSD1-CoREST functions as an allosteric nanoscale binding clamp, which is regulated by peptide substrate binding. This locks LSD1 in a more open but less flexible conformation (Baron and Vellore, 2012). Tranylcypromine derivatives may irreversibly block the normal dynamic flexibility of the complex, preventing engagement with peptide substrates.

Our data suggest that inhibitors of LSD1 may have therapeutic roles in a wider range of malignancies, where disease is consequent upon the activity of SNAG domain transcription factors. For example, GFI1 or GFI1B are oncogenic drivers in medulloblastoma (Northcott et al., 2014), GFI1 represses TP53 in T-acute lymphoblastic leukemia to prevent apoptosis (Khandanpour et al., 2013), and SNAIL family transcription factors are master regulators of the epithelial-to-mesenchymal transition, an essential aspect of cancer cell migration and metastasis (Ferrari-Amorotti et al., 2013, Lamouille et al., 2014). Further, inhibitors of LSD1 may also be effective in combination with other agents: additive or synergistic effects in pre-clinical studies have been reported for tranylcypromine with all-*trans* retinoic acid (Schenk et al., 2012) or SP2509 with panobinostat (Fiskus et al., 2014). Whether a similar mechanism underlies the cellular phenotypes induced by LSD1 inhibitors in all malignant cells remains unclear. In small-cell lung cancer cells, displacement of LSD1 from chromatin following LSD1 inhibition was not observed (Mohammad et al., 2015), whereas in Kasumi-1 cells (McGrath et al., 2016) and in our study, it was. This suggests cell-type-specific differences in cellular mechanism of action.

Interestingly, our study also uncovered a resistance mechanism to tranylcypromine-derivative inhibitors. Mutation of the K661 residue inactivates the catalytic activity of LSD1 through mis-orientation or mis-polarization of FAD (Polticelli et al., 2005) without altering the structural integrity of the protein. Therefore, LSD1 continues to interact with SNAG domain transcription factors to recruit HDACs but cannot demethylate histone tails. An additional consequence of FAD mis-orientation is that tranylcypromine derivatives are no longer able to bind FAD to block both catalytic and structural activities of LSD1. The K661 LSD1 mutant is, therefore, resistant to tranylcypromine-derivative inhibitors. It is possible that reversible inhibitors of LSD1 might be effective alternatives under these circumstances.

We noted that inhibition of LSD1 also mimicked KD of the transcription activator MYB, although the reasons for this remain unclear. Consistent with its role as a transcription activator, we found that MYB-bound enhancers show significantly greater histone acetylation than GFI1-bound enhancers. In contrast to GFI1 and LSD1 binding peaks, however, following treatment of cells with OG86, there was only a modest reduction in the number of MYB binding peaks genome-wide and no change in acetylation of surrounding chromatin. One potential explanation for the shared transcriptional features of LSD1 inhibition and MYB KD is that loss of GFI1:LSD1 from chromatin exposes coincident CEBPA and SPI1 binding sites for occupancy, resulting in upregulation of their target genes. This is in keeping with prior observations that SPI1 can antagonize the activity of MYB at key monocyte/macrophage lineage genes (Reddy et al., 1994) and also the inverse correlation of the transcriptional consequences of LSD1 inhibition versus SPI1 or CEBPA KD in THP1 cells (Suzuki et al., 2009).

In summary, our work reports an unexpected mechanism of action for pharmacologic inhibitors of an important epigenetic target that are already showing promising signs of clinical activity.

## Experimental Procedures

### Human Tissue, Ethical Approvals, and Cell Lines

Use of human tissue was in compliance with the ethical and legal framework of the UK’s Human Tissue Act, 2004. Primary human AML samples were from Manchester Cancer Research Centre’s Tissue Biobank (instituted with the approval of the South Manchester Research Ethics Committee). Their use was authorized following ethical review by the Tissue Biobank’s scientific sub-committee and with the informed consent of the donor. THP1 cells were purchased from DMSZ (Braunschweig, Germany). Details of cell-culture methods are in the Supplemental Information.

### Reagents, Antibodies, and Biochemical Methods

OG86, GSK354, PDD31777, and PDD32116 were synthesized in house, as described previously (Harris et al., 2012, Hitchin et al., 2013, Mould et al., 2017a, Mould et al., 2017b). Details of other reagents, antibodies, and biochemical methods are given in the Supplemental Information.

### RNA-Seq, ChIP-Seq, and Data Analysis

Details are in the Supplemental Information.

### Expression Constructs, Lentiviral Vectors, and Retroviral Vectors

Details of vectors and cloning strategies are given in the Supplemental Information. Lentiviral and retroviral supernatants were prepared, and leukemic human and murine cells were infected with viral particles, as described previously (Harris et al., 2012).

### Flow Cytometry

Flow cytometry analyses were performed using either an LSR Model II BD FACSArray (BD Biosciences, Oxford, UK) or a Novocyte (Acea Biosciences, San Diego, CA, USA) flow cytometer. Cell-sorting experiments were performed using either Influx or FACSAria fluorescence-activated cell sorters (both from BD Biosciences). Antibodies used were anti-human CD11b-PE, anti-human CD14-FITC (fluorescein isothiocyanate), anti-human CD86-PerCP-eFluor710, and anti-Human CD117-PE (eBioscience, Hatfield, UK).

### Statistics

Statistical analyses were performed using Microsoft Excel 2007 or StatsDirect software (v.1.9.7) (StatsDirect, Altrincham, UK).

## Data and Software Availability

The accession number for high-throughput sequencing data files reported in this paper is GEO: GSE63222.

## Supplemental Information

Document S1. STAR Methods, Figures S1–S7, and Tables S1 and S5

Table S2. Gene Expression in THP1 AML Cells Treated with 250 nM OG86 or DMSO Vehicle, Related to Figure 1 Only expressed genes were considered in the analysis (i.e., the 10,002 Human Genome Consortium annotated protein-coding genes exhibiting expression levels of >2.0 reads per kilobase per million mapped reads [RPKM] in at least one of the samples).

Table S3. Active Intergenic Enhancer Regions in THP1 AML Cells and “GLR” Enhancers, Related to Figures 1 and 3 Active enhancers were defined as genomic regions at least 5kB from an annotated gene, identified by BayesPeak as exhibiting co-localized peaks of dimethyl-H3K4 and acetyl-H3K9.

Table S4. Gene Sets, Related to Figures 1 and 2 Gene sets show Human Genome Consortium annotated protein coding genes significantly up or downregulated following knockdown of the indicated genes by comparison with control cells, or addition of phorbol ester (PMA), in THP1 AML cells (*P*≤0.01, unpaired t-test and ≥mean two-fold increase or decrease in expression). Array data were extracted from Suzuki et al. (2009).

Table S6. GSEA, Related to Figure 6

Document S2. Article plus Supplemental Information
