# Supplementary material for: Enhancer Activation by Pharmacologic Displacement of LSD1 from GFI1 Induces Differentiation in Acute Myeloid Leukemia
Source: Cell Rep. 2018 Mar 27;22(13):3641–59. doi: 10.1016/j.celrep.2018.03.012 (PMC5896174; doi:10.1016/j.celrep.2018.03.012)
Supplement: Document S1. STAR Methods, Figures S1–S7, and Tables S1 and S5 [file mmc1.pdf]

Cell Reports, Volume *22*

## Supplemental Information

# Enhancer Activation by Pharmacologic Displacement of LSD1 from GFI1 Induces Differentiation in Acute Myeloid Leukemia

**Alba Maiques-Diaz, Gary J. Spencer, James T. Lynch, Filippo Ciceri, Emma L. Williams, Fabio M.R. Amaral, Daniel H. Wiseman, William J. Harris, Yaoyong Li, Sudhakar Sahoo, James R. Hitchin, Daniel P. Mould, Emma E. Fairweather, Bohdan Waszkowycz, Allan M. Jordan, Duncan L. Smith, and Tim C.P. Somervaille**

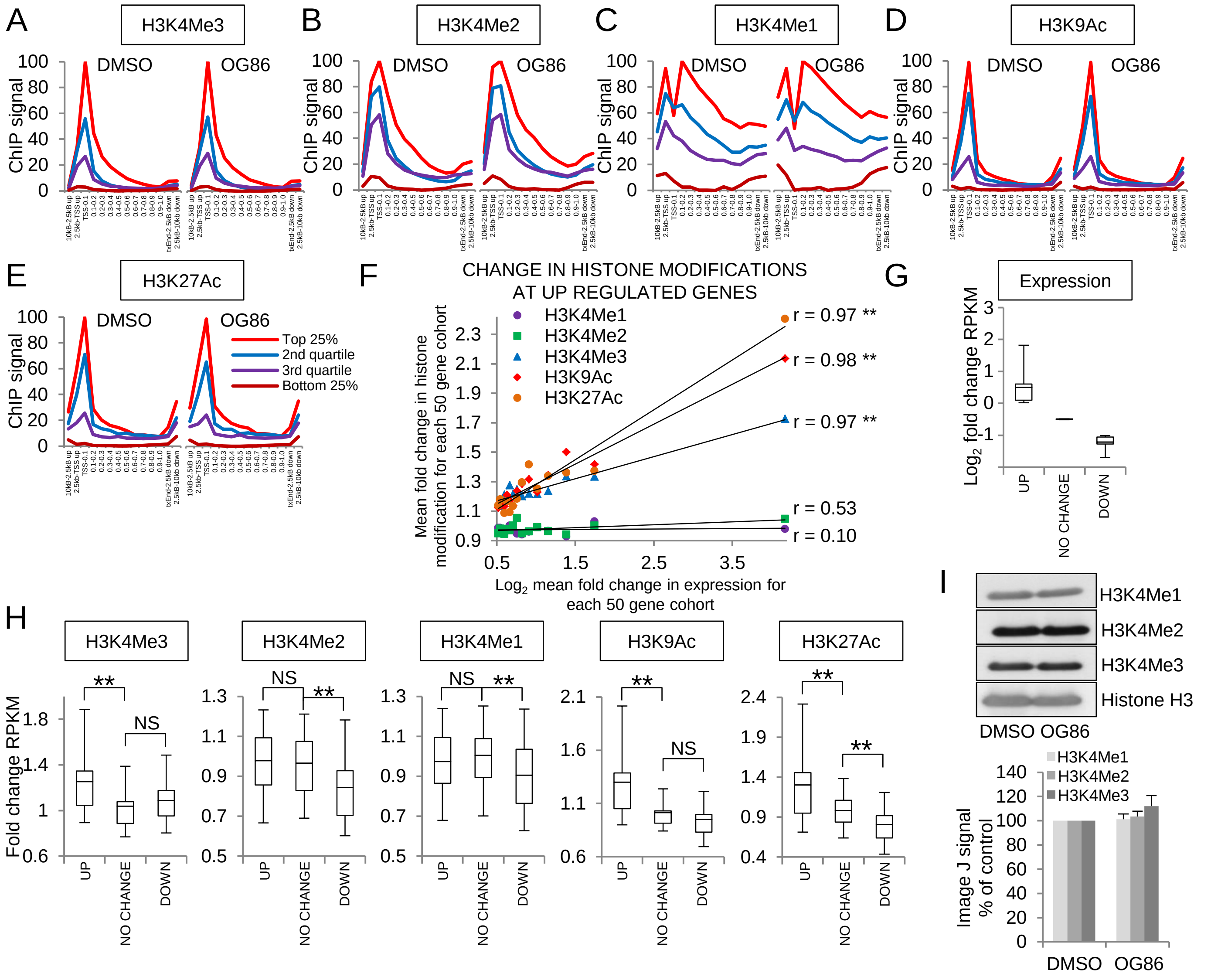


**Figure S1.** Genome wide analysis of histone methylation changes upon pharmacological inhibition of LSD1 with OG86. Related to Figure 1.

(A-H) THP1 AML cells were treated with 250nM OG86 or DMSO vehicle in semi-solid culture for 24 hours prior to RNAseq or ChIPseq. (A-E) Graphs show histone modification profiles across gene bodies and ±10kB. For each graph 18,670 Human Genome Consortium annotated protein-coding genes were ranked according to expression and grouped into quartiles. The open reading frame of genes was divided into ten equal segments for the analysis, with an additional two segments each upstream and downstream as indicated. Individual lines show mean ChIP signal for the indicated cohort of genes at the indicated gene segment. ChIP signal (mean uniquely mapped reads per kilobase per million mapped reads (RPKM)) is normalized to the peak signal for each sample and scaled from 0-100. (F) Up regulated genes were ranked according to fold change in expression (see Table S2) and grouped into cohorts of 50. Graph shows mean fold change in expression ($log_2$ scale) versus mean fold change in the indicated histone modification (RPKM from 2.5kB upstream of the transcription start site (TSS) to midway across gene bodies) for each of 15 cohorts (i.e. 750 genes). The Pearson correlation coefficient, r, for each mark is indicated; ** indicates p≤0.001 (vassarstats.net/rsig.html). (G-H) Box and whisker plots show mean, 25$^{th}$ and 75$^{th}$ centile values (box), and 5$^{th}$ and 95$^{th}$ centile values (whiskers) for fold change in (G) expression (by RNAseq) and (H) ChIP signal for the indicated histone modifications (from 2.5kB upstream of the transcription start site to the midpoint of the coding sequence for each gene). Comparator genes sets are up regulated genes (UP) (n=766; $log_2$ fold increase in expression of 0.5), down regulated genes (DOWN) (n=331; $log_2$ fold decrease in expression of 0.5) and genes whose expression did not change (n=476; fold change in expression <0.015 on the $log_2$ scale). ** indicates p≤0.01 by one-way ANOVA with Tukey's honest significant difference *post hoc* test. NS = not significant. (I) THP1 cells were treated with 250nM OG86 or DMSO vehicle for seven days. Representative western blots (left panel) show global expression of the indicated histone modifications. Bar chart (right panel) shows mean±SEM values for densitometry analysis of western blots using Image J software (n=3).

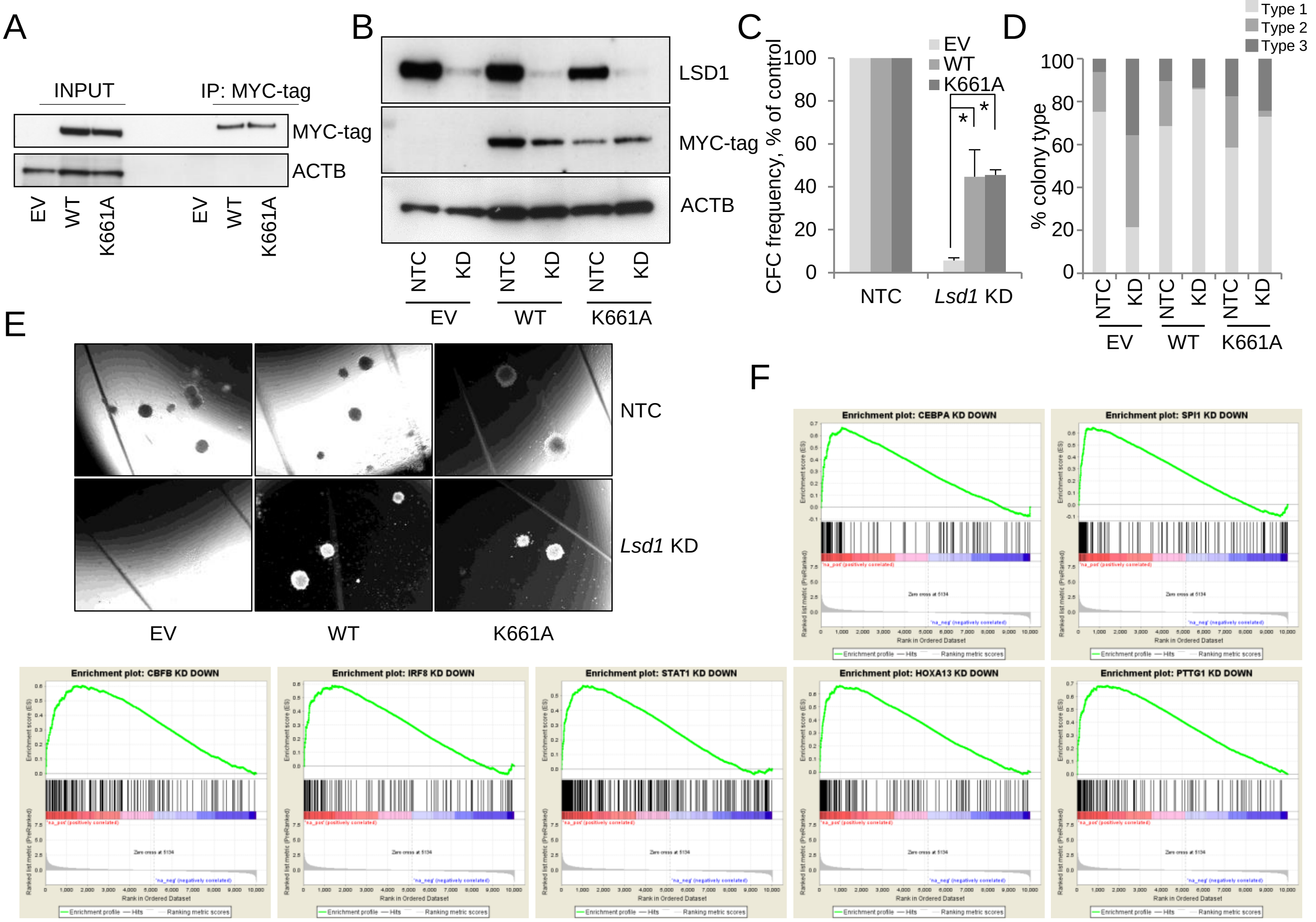


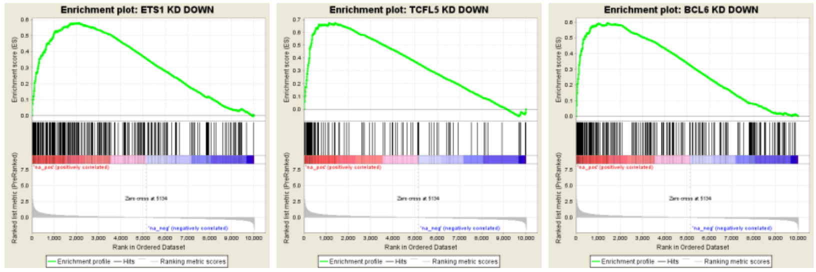


**Figure S2.** LSD1 catalytic activity and AML cell clonogenic potential; gene sets up regulated after LSD1 inhibition. Related to Figure 2 and Tables S3 and S4

(A) 293FT cells were transfected with an empty retroviral vector (EV) or vectors expressing MYC-tagged wild type (WT) or K661A mutant LSD1. 48 hours later cells were lysed and lysates immunoprecipitated with a MYC-tag antibody. Western blots show expression of tagged proteins and the washed immune complexes used in an *in vitro* LSD1 demethylation assay (see also Figure 2A). (B-E) Murine MLL-AF9 AML cells were infected with retroviruses expressing MYC-tagged WT or K661A mutant LSD1, or an EV, with GFP as the selectable marker. FACS-purified $GFP^{+}$ cells were then infected with lentiviruses expressing an shRNA targeting *Lsd1* for knockdown (KD) or a non-targeting control (NTC), with puromycin drug resistance as the selectable marker. (B) Western blot shows expression of the indicated proteins in the indicated conditions 48 hours following initiation of drug selection. (C) Bar chart shows mean±SEM colony forming cell (CFC) frequencies of OG86-resistant cells relative to controls, enumerated after six days in semisolid culture (n=3). * indicates $P<0.05$ for the indicated comparison using one-way ANOVA and Fisher's least significant difference *post hoc* test. (D) Bar chart indicates the proportion of colonies of the indicated type in each condition from a representative experiment from (C). Type 1 colonies contain poorly differentiated myeloblasts, Type 2 colonies contain a mixed population of blasts and differentiating myeloid cells and Type 3 colonies contain terminally differentiated macrophages (Harris et al., 2012). (E) Representative images of murine MLL-AF9 AML cell colonies after six days in semi-solid culture. (F) GSEA plots show enrichment of gene sets down regulated following KD of the indicated transcription factor (Suzuki et al., 2009) among genes up regulated following treatment of THP1 AML cells with 250nM OG86 for 24 hours.

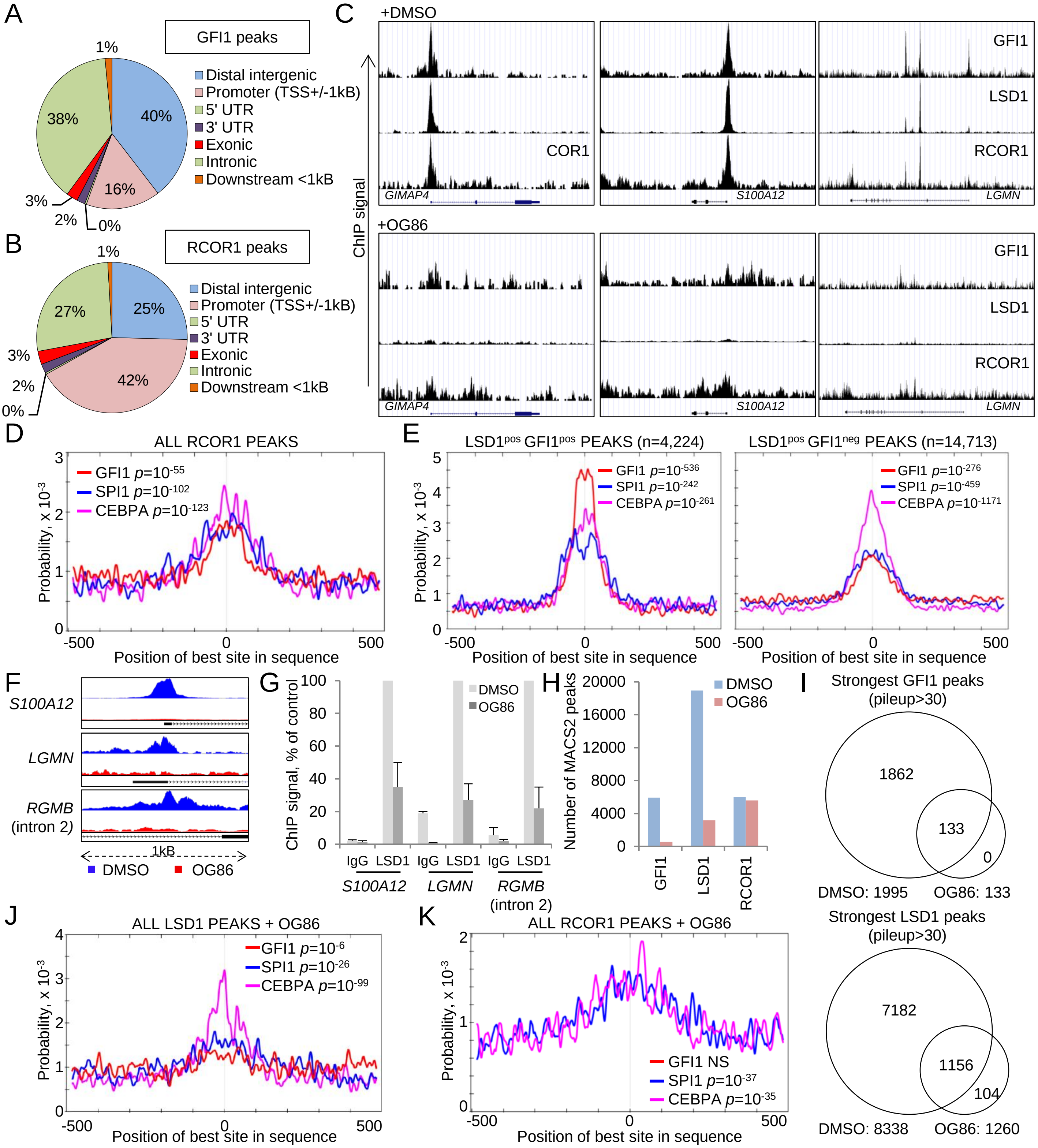


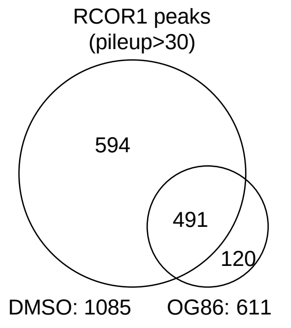


**Figure S3**. Interaction of LSD1 with chromatin after OG86 exposure. Related to Figure 3.

(A & B) Pie charts indicates genome location annotations for 5,924 GFI1 and 5,980 RCOR1 binding peaks in THP1 AML cells. (C) Representative ChIPseq tracks (ChIP signal = reads per kilobase per million mapped reads, RPKM). (D, E, J & K) MEME-ChIP motif enrichment plot with indicative *P* values for the indicated sets of binding peaks. (F&G) Representative LSD1 ChIPseq tracks at promoters (*S100A12* and *LGMN*) and an intronic enhancer (*RGMB*) (F) with (G) ChIP PCR analysis using primers targeting the loci indicated in (F). Error bars refer to SEM; n=3. (H) Total binding peaks. (I) Intersection analysis of the strongest GFI1, LSD1 and RCOR1 peaks in vehicle versus OG86-treated cells.

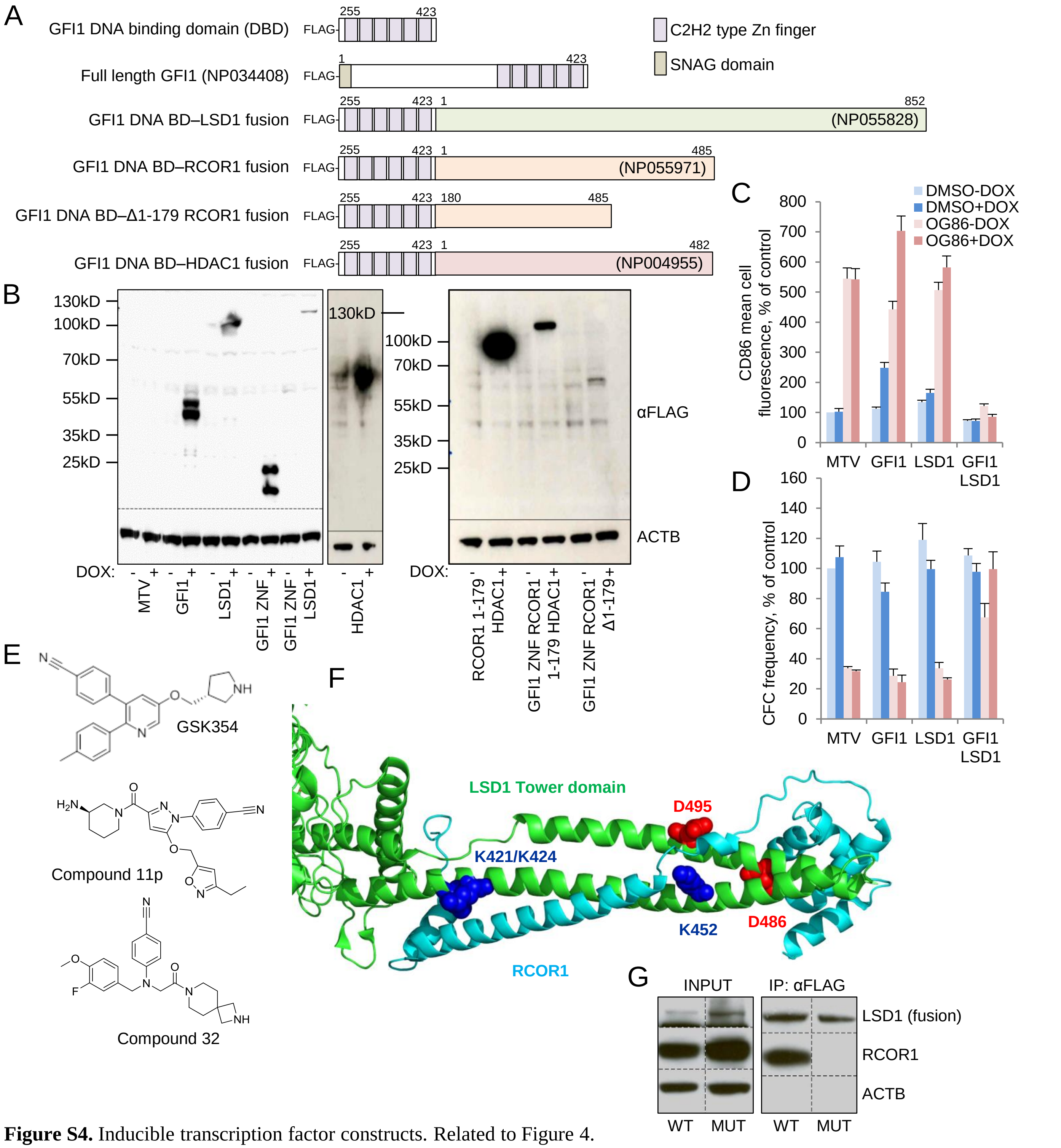


**Figure S4.** Inducible transcription factor constructs. Related to Figure 4.

(A) Images show domain structure of GFI1, LSD1, RCOR1 and HDAC1 constructs expressed using lentiviral vectors. (B) Western blots show doxycyline-induced expression of the indicated protein and transcription factors constructs in THP1 AML cells. (C-D) THP1 AML cells infected with lentiviruses expressing GFI1 fusion or control constructs regulated by a doxycycline-regulated promoter were treated with 250nM OG86 or DMSO vehicle in the presence or absence of doxycycline. Bar charts indicate (C) mean±SEM CD86 mean cell fluorescence 24 hours later, as determined by flow cytometry, in the indicated conditions (n=3 for each panel) and (D) mean±SEM colony forming cell (CFC) frequency (n=3 for each panel). Colonies were enumerated ten days later. MTV = empty vector. (E) LSD1 inhibitor structures. (F) Image shows location of mutated LSD1 Tower domain residues on the X-ray crystal structure of the LSD1:CoREST complex (PDB code 2XAS). (G) Following induced expression of the wild type (WT) GFI1 ZNF LSD1 fusion or a K421D/K424D/K452D/D495K quadruple mutant (MUT), anti-FLAG immunoprecipitations were performed. Western blots for the indicated proteins are shown.

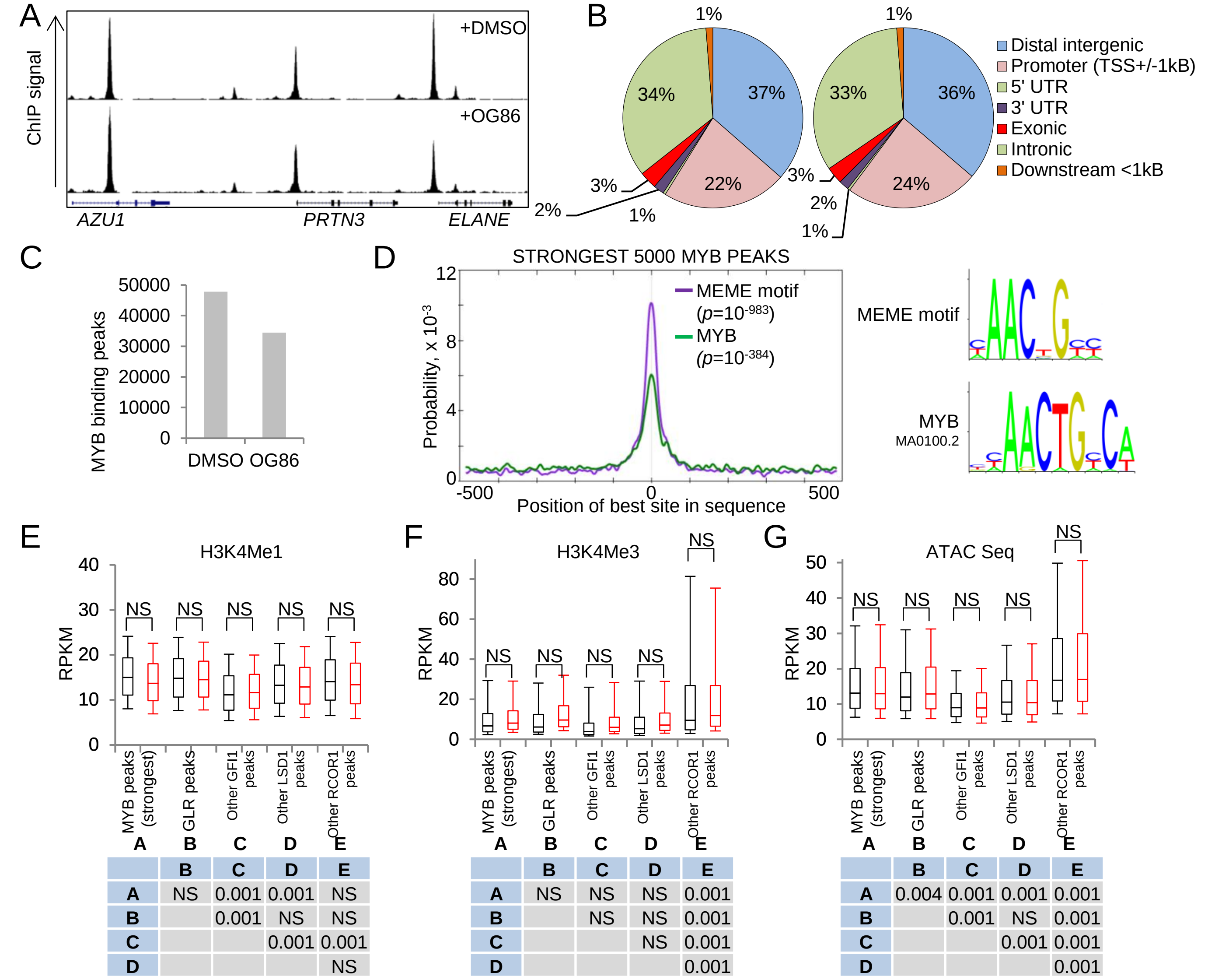


E (H3K4Me1):

| | B | C | D | E |
|---|---|---|---|---|
| A | NS | 0.001 | 0.001 | NS |
| B | | 0.001 | NS | NS |
| C | | | 0.001 | 0.001 |
| D | | | | NS |

F (H3K4Me3):

| | B | C | D | E |
|---|---|---|---|---|
| A | NS | NS | NS | 0.001 |
| B | | NS | NS | 0.001 |
| C | | | NS | 0.001 |
| D | | | | 0.001 |

G (ATAC Seq):

| | B | C | D | E |
|---|---|---|---|---|
| A | 0.004 | 0.001 | 0.001 | 0.001 |
| B | | 0.001 | NS | 0.001 |
| C | | | 0.001 | 0.001 |
| D | | | | 0.001 |

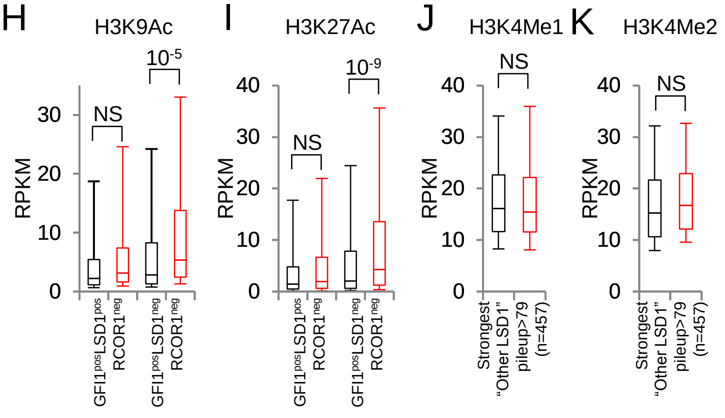


**Figure S5.** MYB ChIPseq signal, & histone modifications surrounding MYB, GFI1, LSD1 and RCOR1 enhancer binding sites following LSD1 inhibition. Related to Figure 5.

THP1 AML cells were treated with 250nM OG86 or DMSO vehicle for 48 hours. (A) Exemplar ChIPseq tracks for MYB. (B) Pie charts indicate genome location annotations for 47,818 (DMSO vehicle: left panel) or 34,426 (OG86; right panel) MYB binding peaks. (C) Graph shows number of MYB binding peaks in the indicated conditions. (D) MEME-ChIP motif enrichment plots with indicative *P* values for the strongest 5,000 MYB binding peaks. (E-G) Box and whisker plots show median, 25$^{th}$ and 75$^{th}$ centile values (box), and 10$^{th}$ and 90$^{th}$ centile values (whiskers) for normalized ChIP or ATACseq signal for (E) H3K4Me1, (F) H3K4Me3 and (G) ATACseq signal surrounding (±1kB) the indicated sets of binding peaks. Black boxes = DMSO vehicle; red boxes = OG86. Indicative *P* values (t-test) are shown for the indicated comparisons. NS = not significant. Tables beneath box and whisker plots show *P* values for comparisons of signal at the indicated sets of binding peaks (labelled A-E) in the DMSO vehicle condition, as determined by one way ANOVA and Tukey's honest significant difference *post hoc* test. (H-K) Box and whisker plots show median, 25$^{th}$ and 75$^{th}$ centile values (box), and 10$^{th}$ and 90$^{th}$ centile values (whiskers) for normalized ChIP signal for (H) H3K9ac, (I) H3K27ac, (J) H3K4me1 and (K) H3K4me2 signal surrounding (±1kB) the indicated sets of binding peaks. Black boxes = DMSO vehicle; red boxes = OG86. Indicative *P* values (t-test) are shown for the indicated comparisons. NS = not significant.

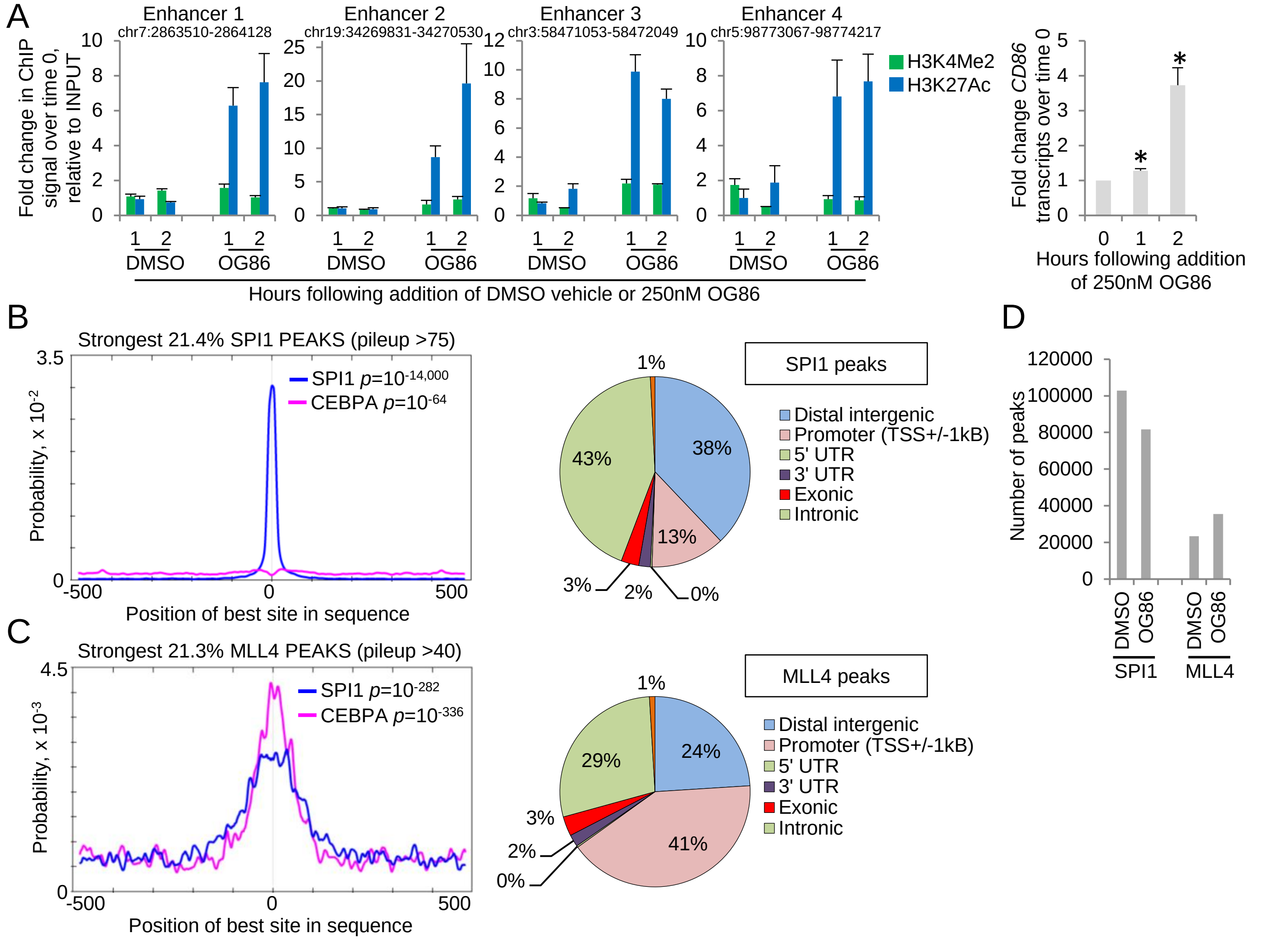


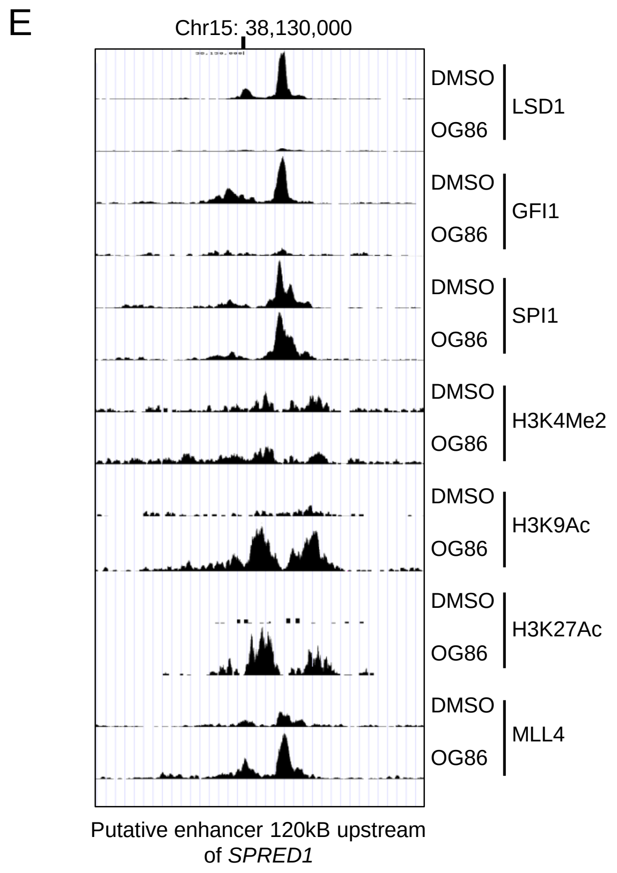


**Figure S6.** Timecourse of increased enhancer acetylation; SPI1 and MLL4 ChIPseq. Related to Figure 6.

(A) THP1 AML cells were treated with 250nM OG86 or DMSO vehicle and ChIP-PCR was performed for the indicated histone modifications at the indicated times and using primers for the indicated putative enhancers (genomic coordinates are for hg38 build). Graphs (left panels) show mean±SEM ChIP signal (n=3). Right panel: *CD86* expression. * indicates *P*<0.05 by one way ANOVA and Fisher's least significant difference post hoc test. (B & C) MEME-ChIP motif enrichment plots with indicative *P* values (left panels) and pie charts indicating genome location annotations (right panels) for SPI1 and MLL4 binding peaks in THP1 AML cells. (D) Total binding peaks in control and OG86-treated THP1 AML cells. (E) Indicative ChIP seq tracks.

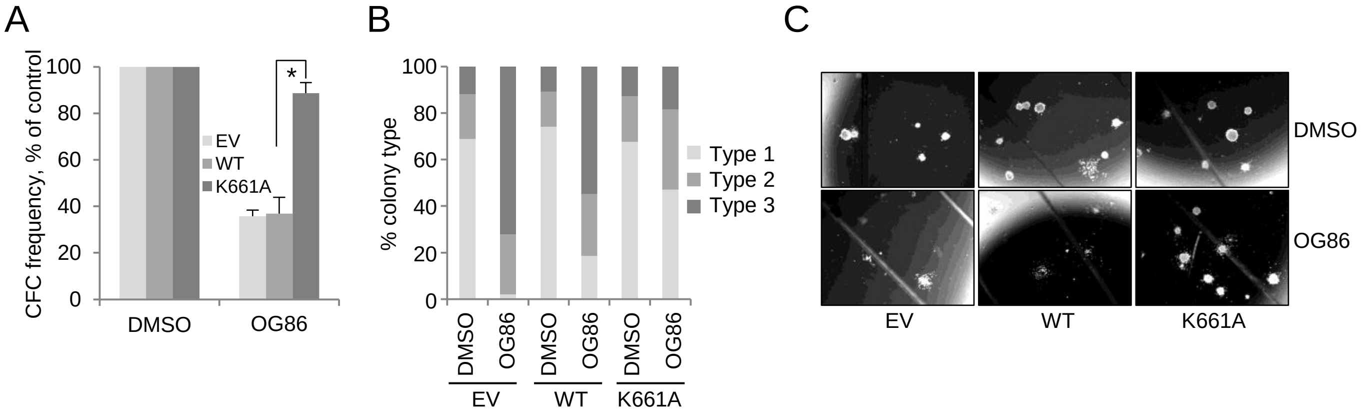


**Figure S7.** A K661A LSD1 mutation renders murine MLL-AF9 AML cells resistant to OG86. Related to Figure 7.

Murine MLL-AF9 AML cells were infected with retroviruses expressing MYC-tagged wild type (WT) or K661A mutant LSD1, or an empty vector (EV), with GFP as the selectable marker. FACS-purified $GFP^+$ cells were treated with 250nM OG86 or DMSO vehicle control. (A) Bar chart shows mean±SEM colony forming cell (CFC) frequencies relative to control cells for the indicated lines enumerated after six days in semisolid culture (n=3). * indicates $P<0.05$ for the indicated comparison using one-way ANOVA and Fisher's least significant difference *post hoc* test. (B) Bar chart indicates the proportion of colonies of the indicated type in each condition from a representative experiment from (A). Type 1 colonies contain poorly differentiated myeloblasts, Type 2 colonies contain a mixed population of blasts and differentiating myeloid cells and Type 3 colonies contain terminally differentiated macrophages (Harris et al., 2012). (C) Representative images from (A).

## Supplemental Tables

**Table S1.** Karyotypes of primary AML samples. Related to Figure 1.

| Biobank number | BM or PB | Karyotype |
|---|---|---|
| 104 | BM | 46,XX,t(6;9;11)(p2?1;p22;q23)[6]/ 45,idem,der(15)t(15;17)(p11.2;q11.2),-17[4] |
| 108 | BM | 46,XX,t(6;11)(q27;q23)[10] |
| 148 | PB | 46,XY,t(6;11)(q27;q23)[10]/ 48,idem,+der(6)t(6;11),+21[4] |
| 160 | PB | 46,XX,t(9;11)(p22;q23),der(21;22)(q10;q10),+der(21;22)[10] |
| 419 | PB | 46,XX,t(1;22)(p21;p11.2),ins(10;11)(p12;q23q1?4)[10] (MLL gene rearrangement confirmed by FISH) |
| 514 | BM | 46,XX,t(9;11)(p22;q23)[1]/ 46,XX[7] |
| 518 | BM | 45,XY,der(10)t(10;11)(p1?2;q23),der(10;12)(q10;q10),der(11)t(10;11)inv(11)(q21q23)[7]/ 45,XY,?add(7)(q?22),?add(10)(p?11.2),der(10;12)(q10;q10),der(11)t(10;11)inv(11)(q21q23), ?i(17)(q10)[3] |
| 582 | BM | 46,XX,der(1)t(1;1)(p36;q25),t(5;9;11)(q3?3;p22;q23)[5]/ 46,XX,t(5;9;11),add(17)(p11.2)[3]/46,XX,t(5;9;11),add(12)(q24)[2] |

**Table S5.** Gene sets significantly enriched among genes up or down regulated following treatment of THP1 AML cells with OG86. Related to Figure 2.

Significant enrichment is defined as false discovery rate (FDR) ≤5%, p≤0.01, normalized enrichment score (NES)>2 with a gene set size >75 (Subramanian et al., 2005).

| *Enriched in genes up regulated by LSD1 inhibition* | | | | *Enriched in genes down regulated by LSD1 inhibition* | | |
|---|---|---|---|---|---|---|
| **Gene set** | **NES** | **FDR (%)** | | **Gene set** | **NES** | **FDR (%)** |
| GFI1 KD UP | 2.7 | 0 | | GFI1 KD DOWN | -2.4 | 0 |
| MYB KD UP | 2.6 | 0 | | MYB KD DOWN | -2.2 | 0 |
| PTTG1 KD DOWN | 2.3 | 0 | | | | |
| HOXA13 KD DOWN | 2.2 | 0 | | | | |
| TCFL5 KD DOWN | 2.2 | 0 | | | | |
| SPI1 KD DOWN | 2.2 | 0 | | | | |
| CEBPA KD DOWN | 2.1 | 0 | | | | |
| BCL6 KD DOWN | 2.1 | 0 | | | | |
| ETS1 KD DOWN | 2.1 | 0 | | | | |
| CBFB KD DOWN | 2.1 | 0 | | | | |
| STAT1 KD DOWN | 2 | 0 | | | | |
| IRF8 KD DOWN | 2 | 0 | | | | |

## Supplemental Experimental Procedures

### Reagents and antibodies

Reagents were: doxycycline (Clontech, Mountain View, CA), vorinostat, JQ1+ and JQ1- (all from Sigma Aldrich, Gillingham, UK). Antibodies for western blotting were: anti-LSD1 (ab17721), anti-HDAC1 (ab46985) (both from Abcam, Cambridge, UK), anti-ACTB (MAB1501), anti-RCOR1 (07-455) (both from Merck Millipore, Billerica, MA), anti-HDAC2 (sc-7899), anti-GFI1 (sc-8558) (both from Santa Cruz Biotechnology, Dallas, TX), anti-Myc tag (2276), anti-monomethyl H3K4 (9723), anti-dimethyl H3K4 (9725), anti-trimethyl H3K4 (9727), anti-histone H3 (3638) (all from Cell Signaling Technology, Danvers, MA) and anti-FLAG (F3165; Sigma Aldrich). All were used at a dilution of 1:1000 except anti-ACTB (1:10,000), anti-Myc tag (1:2000) and anti-GFI1 (1:200). Antibodies used for immunoprecipitation

experiments were as above and: IgG Rabbit (12-307), IgG Mouse (12-371) and IgG Goat (NI02) (all from Merck Millipore).

**Cells and cell culture**

THP1 cells were cultured in RPMI 1640 with 10% fetal bovine serum (FBS) or methylcellulose (H4320, Stem Cell Technologies, Vancouver, BC). Murine MLL-AF9 AML cells, generated using a retroviral transduction and transplantation approach, were recovered from sick mice and cryopreserved as described (Harris et al., 2012). Following thawing, cells were cultured in RPMI 1640 containing 20% FBS with 5% X63 supernatant (Karasuyama and Melchers, 1988) or methylcellulose medium (M3231, Stem Cell Technologies) containing 20ng/ml SCF, 10ng/ml IL6, 10ng/ml GM-CSF and 10ng/ml IL3 (Peprotech, London, UK). Culture densities were $5x10^4$ - $5x10^5$ for cells in liquid culture. For semisolid culture, starting culture density was $10^3$/ml. Colonies were enumerated 5-10 days later.

Cryopreserved leukemic blast cells from BM or blood of patients at presentation were thawed and co-cultured on MS5 stromal cells in α-MEM medium supplemented with 12.5% heat-inactivated FBS, 12.5% heat-inactivated horse serum, 2mM L-glutamine, 57.2µM β-mercaptoethanol, 1µM hydrocortisone and IL3, G-CSF and TPO (all at 20ng/ml; Peprotech) for seven days to allow for recovery from cryopreservation. Cells were then transferred to fresh stromal layers and cultured for a further seven days in OG86 250nM or DMSO control. In *GFI1* KD experiments (see below), cells were cultured overnight in viral supernatant supplemented with IL3, G-CSF and TPO (all at 20ng/ml), transferred to stromal layers and then cultured for a further three days prior to analysis. Leukemia cells (single cells) were readily separated from stromal cells (adhesive clumps) through disruption of the stromal layer by pipetting and then filtering the whole through a 75µm filter basket.

**RNA sequencing and data analysis**

Total RNA was extracted from DMSO vehicle or OG86-treated THP1 AML cells using QIAshredder spin columns and an RNeasy Plus Micro Kit (Qiagen, Manchester, UK). PolyA selection using 15µg total RNA was carried out by performing three rounds of selection using a MicroPoly(A)Purist Kit. Barcoded polyA libraries for pooling and sequencing were prepared using 55ng of the polyA selected RNA with a SOLiD Total RNAseq Kit. Following quantitation of the libraries by Q-PCR using a SOLiD Library TaqMan Quantitation Kit, emulsion PCR was performed using the SOLiD EZBead System prior to sequencing of single-ended strand-specific 50mers using a SOLiD 5500 System (all from Life Technologies, Paisley, UK).

Reads were aligned to the human genome (build hg19) with SHRIMP2 (Langmead et al., 2009; David et al., 2011) using default settings. Reads aligning to multiple loci were discarded. There were 58.5 million and 67.6 million uniquely mapped reads for the DMSO and OG86 treated THP1 cell samples respectively. Data from two technical replicates for each sample were merged. 90.8% and 90.5% of reads mapped to annotated protein coding genes (ENSEMBL v66) using the Annmap database, R and

Bioconductor (Gentleman et al., 2004; Yates et al., 2007). RPKM (reads per kilobase per million uniquely mapped reads) was computed for each transcript. Gene level expression values were calculated as the mean RPKM expression for all transcripts arising from the same annotated gene. Genes annotated as protein coding in ENSEMBL v66 but not by the Human Genome Consortium (HGNC) (www.genenames.org, access date 6 June 2016) were discarded, as were mitochondrial genes, leaving 18670 for analysis. Once genes with expression levels less than 2 RPKM in both samples were discarded, 10,002 remained for downstream analyses (Table S2). Data files are available at the Gene Expression Omnibus: GSE63222.

**Gene set enrichment analysis**

Pre-ranked gene set enrichment analysis was performed with GSEA v2.0.14 software from www.broadinstitute.org/gsea (Subramanian et al., 2005). Genes were rank ordered according to $\log_2$ fold change in expression (Table S2). For gene sets from the FANTOM Consortium (Suzuki et al., 2009), normalized array data were downloaded (fantom.gsc.riken.jp/4). For each transcription factor or other gene where array data confirmed knockdown (n=46), expressed HGNC-annotated protein coding genes were identified that exhibited (i) significantly different expression levels (i.e. $P \leq 0.01$, unpaired t-test) and (ii) at least a mean 2-fold increase or decrease in expression in knockdown cells by comparison with control cells. Genes were deemed expressed where the mean array expression value of either control or knockdown samples was ≥30. An identical approach was used to identify genes differentially regulated by a 24-hour treatment of THP1 cells with PMA (Suzuki et al., 2009). Gene sets are shown in Table S4.

**Chromatin immunoprecipitation and next generation sequencing**

ChIPs for methyl histone modifications were performed using a HighCell# ChIP kit (Diagenode, Liege, Belgium) according to manufacturer's instructions. Antibodies used were: anti-monomethyl H3K4 (C15410037; 1.7ul per ChIP), anti-dimethyl H3K4 (C15410035; 1.7ul per ChIP) and anti-trimethyl H3K4 (C15410003; 2ul per ChIP) (all from Diagenode). ChIPs for acetyl-H3K9 (ab4441; 5ul per ChIP) and acetyl-H3K27 (ab4729; 5.6ul per ChIP) (both from Abcam) were performed using 50 million cells and the protocol of Lee et al. (2006). Prior to ChIPseq, DNA was purified with an iPure kit (Diagenode), according to manufacturer's instructions.

To prepare samples for sequencing on the Illumina HiSeq 2500 (Illumina, San Diego, CA), a Microplex Library Preparation Kit (Diagenode) was used to generate libraries from 1ng ChIP DNA. Libraries were then size selected (200-800 base pairs) by adding 0.55x volume of AMPure beads (Beckman Coulter, Pasadena, CA) followed by 0.3x volume of AMPure beads to the supernatant. The supernatant was then discarded and the beads washed with 70% ethanol before drying and elution of the size selected library. Library quantitation was performed by Q-PCR using a KAPA Library Quantification Kit (Kapa Biosystems, Woburn, MA). Next, 15pM of the library was used for on board cluster generation

in the Rapid Mode of a HiSeq 2500 (Illumina) and then paired end 75 or 101 base pair sequencing was performed using a TruSeq Rapid SBS Kit (Illumina).

For ChIP for MYB, GFI1, LSD1, RCOR1, SPI1 and MLL4 THP1 cells were cultured for 48 hours in the presence of DMSO or OG86 at a density of $3x10^5$/ml. Cells were cross-linked at room temperature using 1% formaldehyde. After 10 minutes the reaction was stopped by incubation for five minutes with 0.125M glycine. Cell pellets were washed twice with cold PBS containing protease inhibitors (Complete EDTA-free tablets, Roche, Basel, Switzerland). 100 million cells were used per ChIP, as described (Lee et al., 2006). Briefly, nuclear lysates were sonicated using a Bioruptor Plus (Diagenode) for 15 min at high, 30 sec ON, 30 sec OFF settings. Immunoprecipitation was performed overnight at 20rpm and 4°C, with 100µl magnetic beads (Dynabeads (Protein G), Invitrogen, Carlsbad, CA) per 10µg antibody. Antibodies were: LSD1 (ab17721), GFI1 (ab21061) and MYB (ab45150) (all from Abcam), RCOR1 (07-455 from Merck Millipore), SPI1 (2258 from Cell Signaling) and MLL4 (kindly provided by Dr Kai Ge; Wang et al., 2016). After washing six times with RIPA buffer (50mM HEPES pH 7.6, 1mM EDTA, 0.7% Na deoxycholate, 1% NP-40, 0.5M LiCl), chromatin IP-bound fractions were extracted at 65°C for 30min with elution buffer (50mM TrisHCl pH8, 10mM EDTA, 1% SDS) vortexing frequently. RNAseA (1mg/ml) and proteinase K (20mg/ml) were used to eliminate any RNA or protein from the samples. Finally DNA was extracted using phenol:chloroform:isoamyl alcohol extraction and precipitated with ethanol (adding two volumes of ice-cold 100% ethanol, glycogen (20µg/µl) and 200mM NaCl) for at least 1 hour at -80°C. Pellets were washed with 70% ethanol and eluted in 50µl 10mM TrisHCl pH8.0.

ChIP DNA samples were prepared for sequencing using the Microplex Library Preparation Kit (Diagenode) and 1ng ChIP DNA. Libraries were size selected with AMPure beads (Beckman Coulter) for 200-800 base pair size range and quantified by Q-PCR using a KAPA Library Quantification Kit. ChIPseq data were generated using the NextSeq platform from Illumina with 2x75bp Mid Output.

Reads were aligned to human genome hg38 using BWA-MEM (version 0.7.13) (http://bio-bwa.sourceforge.net/, using 16 threads and with -M set to flag shorter split hits as secondary) or Bowtie2 (version 2.2.1) (http://bowtie-bio.sourceforge.net/bowtie2/) using default settings. Reads were then filtered using Samtools (version 0.1.9) (Li et al., 2009) keeping only reads with alignment quality score >= 20. The number of uniquely mapped reads per sample was 50-100 million. Reads were mapped relative to annotated genes (ENSEMBL v66) using the Annmap database, R and Bioconductor (Gentleman et al., 2004; Yates et al., 2007). MACS2 (Model-based Analysis of ChIP-seq, version 2.1.0) software was used to call peaks (Zhang et al., 2008). DMSO- or OG86-treated input samples were respectively used as reference, duplicates at the exact same location were removed and a cutoff of 0.01 False Discovery Rate (FDR) was used as a threshold. Only those peaks showing pileup values ≥18 and log(q value) ≥3 were deemed to have met threshold criteria and were considered for further analysis. Using the ChIPseeker package (version 1.10.3) (R, Bioconductor) (Yu et al., 2015) peak coordinates were annotated to the nearest genomic features using transcript-related features from UCSC hg38. Transcript start site (TSS) region was defined as ±1kb from the TSS. As some peaks overlap multiple genomic regions, the package

adopted the following priority in annotation: promoter, 5' UTR, 3' UTR, exon, intron, downstream, intergenic.

A ChIPpeakAnno package (version 3.8.9) (R/Bioconductor) (Yu et al., 2015) was used to find peaks with apices located within 500bps of one another and to extract sequences in FASTA format around the summit of each peak. For motif analysis a window of ±500bp around the summit was analyzed using MEME-ChIP (version 4.12.0) (Machanick & Bailey, 2011) with default parameters. The genomic coordinates of peak apices were set at the centers of 500bp regions to create a BED file using the package GenomicRanges (version 1.30.1) (R/Bioconductor) (Lawrence et al., 2013). Then they were used for evaluating the intersection of peaks between different ChIPseq experiments with the BEDtools package (version 2.25.0) (Quinian and Hall, 2010).

For analysis of histone marks, for each gene the gene body (i.e. from the transcription start site (TSS) to the end of the gene) was divided into ten sub-regions of equal length. The region upstream of the gene was divided into two regions: from 10kb to 2.5kb upstream, and from 2.5kb upstream to the TSS. The region downstream of the gene was similarly divided. Therefore each gene consists of 14 regions covering upstream sequences, the gene body and downstream sequences. The number of reads mapped to each of the 14 regions for each gene was calculated, as were values for reads per kilobase. For promoter analyses, and for analyses surrounding transcription factor binding peaks, the region ±2.5kB surrounding the transcription start site or the apex of the transcription factor binding peak was divided into 50 100 base pair sub-regions. The number of reads mapped to each of the 50 regions was calculated. Data files are available at the Gene Expression Omnibus: GSE63222.

**ATAC sequencing**

The Assay for Transposase Accessible Chromatin (ATACseq) protocol (Buenrostro et al., 2013) was performed using 50,000 THP1 cells cultured for 24 hours in the presence of DMSO or 250nM OG86 at a density of $3x10^5$/ml. Cell pellets were re-suspended in 50µl lysis buffer (10mM Tris-HCL pH7.4, 10mM NaCl, 3mM $MgCl_2$, 0.1% IGEPAL CA-630) and nuclei were pelleted by centrifugation for 10 minutes at 500g. Supernatant was discarded and the nuclei were re-suspended in 25µl reaction buffer containing 2µl of Tn5 transposase and 12.5µl TD buffer (Nextera DNA Sample Preparation Kit; Illumina). The reaction was incubated for 30 minutes at 37ºC and 300rpm, and purified using the Qiagen MinElute Kit. Library fragments were amplified using 1x NEB Next High-Fidelity PCR master mix and 1.25µM of custom PCR primers and conditions (Buenrostro et al., 2013). The PCR reaction was monitored to reduce GC and size bias by amplifying the full libraries for five cycles and taking an aliquot to run for 20 cycles using the same PCR cocktail and 0.6x SYBR Green. The remaining 45µl reaction was amplified for additional cycles as determined by qPCR. Libraries were finally purified using a Qiagen MinElute Kit. Libraries were size selected with AMPure beads (Beckman Coulter) for 200-800 base pair size range and quantified by Q-PCR using KAPA Library Quantification Kit. ATACseq data were generated using the NextSeq platform from Illumina with a 2x75bp High Output.

Sequencing reads were quality checked using FASTQC (version 0.11.3) (Andrews, 2010). Any adapter sequences present in the data were removed using Cutadapt (version 1.10) (Martin, 2012). The cleaned and trimmed FASTQ files were mapped to the hg38 reference assembly using BWA (version 0.7.13) (Li and Durbin, 2009) and processed using Samtools (version 0.1.9) (Li et al, 2009). The data were cleaned for duplicates, low mapping quality reads (i.e. MAPQ<30), non-uniquely mapped reads, not properly paired reads and reads mapped to non-conventional chromosomes and mitochondrial DNA. Data files are available at the Gene Expression Omnibus: GSE63222.

**ChIP PCR**

For ChIP quantitative PCR, assays were performed in 384-well MicroAmp optical reaction plates using Taqman Fast Universal PCR Mastermix (Life Technologies) and Universal Probe Library System designed primers and probes (Roche). Signal was detected using an ABI PRISM 7900HT Sequence Detection System (Life Technologies). Primers and probes used were:
(i) *S100A12* (F: gagggcaaaattcagtctgg; R: aagcttcaaacctgtggcttag; probe 26);
(ii) *LGMN* (F: aggtgcagaatggtttggaa; R: catctataggaacggcaccaa; probe 81); and
(iii) *RGMB* (F: ctgacgtgcggtcacaac; R: tccacgacgccataatcc; probe 69).

For the timecourse study, THP1 cells were treated for 1 or 2 hours with 250nM OG86 or DMSO vehicle at a cell density of 300,000/ml. At each time point cells were cross-linked and ChIPs were performed as described above using 5 million cells per antibody and time point. Primers and probes used were:
Enhancer 1: F: gccccaacaagtaacctcct ; R: gtgcagaccaccagaggaa; probe 36
Enhancer 2: F: ggagcctagtctgctctctcag; R:gaggaaggagggtgccata; probe 30
Enhancer 3: F: aacctctaaccatggtctttgc; R: gggcaactcagtgaatacgtg; probe 60
Enhancer 4: F: ggaagaagaggaagcgaagc; R: cgtccatgcaggtctcgt; probe 70

**Active intergenic enhancer analysis**

To identify active enhancers in human THP1 AML cells, ChIPseq data sets generated using anti-dimethyl H3K4 and anti-acetyl H3K9 were analysed. Peaks of H3K4 dimethylation and H3K9 acetylation were called using the Bioconductor package BayesPeak (Spyrou et al., 2009). Active enhancers were defined as non-centromeric loci at least 5kB from the coding sequence of any gene with a coincident (i.e. overlapping) peak of H3K4Me2 and H3K9Ac. The ENSEMBL human gene annotation database version 66 was used.

**Protein extraction, western blotting and immunoprecipitation**

For western blotting, cells were lysed in a high salt lysis buffer (45mM HEPES (pH7.5), 400mM NaCl, 1mM EDTA, 10% glycerol, 0.5% Nonidet P-40, 6.25mM NaF, 20mM β-glycerophosphate, 1mM DTT, 20mM sodium orthovanadate and 1x Protease Inhibitor Cocktail (Roche)) and equal amounts of protein

were loaded and separated by SDS-PAGE. For histone western blots, lysates were prepared using a histone acid extraction protocol (Abcam). For subcellular fractionation experiments, lysates were prepared using a Subcellular Protein Fractionation Kit for Cells, according to manufacturer's instructions (Pierce, Rockford, IL). For co-immunoprecipitation of whole cell extracts, cells were lysed in TNN Buffer (50mM Tris-HCl (pH7.5), 100mM NaCl, 5mM EDTA, 0.5% Nonidet P-40, 6.25mM NaF, 20mM β-glycerophosphate, 1mM DTT, 20mM sodium orthovanadate, 1x Protease Inhibitor Cocktail (Roche), 10uM N-ethylmaleimide and 1:10,000 benzonase (Sigma Aldrich)) and lysate was incubated with the appropriate antibody pre-bound to Protein G–Sepharose (Sigma Aldrich) for two hours. Immune complexes were pulled down and washed four times with TNN buffer, eluted and separated by SDS-PAGE. For co-immunoprecipitation of nuclear protein extracts the Nuclear Extraction Protocol (Invitrogen) was used. Briefly, cells were lysed in hypotonic buffer (20mM Tris-HCL, pH7.4, 10mM NaCl, 3mM $MgCl_2$ containing 1x Protease Inhibitor Cocktail (Roche)) and 10% NP40 was added followed by centrifugation for 10 mins at 3000rpm at $4^oC$. The nuclear fraction pellet was resuspended in cell extraction buffer (Invitrogen) (with 6.25mM NaF, 20mM β-glycerophosphate, 1mM DTT, 20mM sodium orthovanadate, 1x Protease Inhibitor Cocktail (Roche) and 1:10,000 benzonase) for 30min on ice followed by centrifugation for 30min at 14,000 x g at 4ºC. The nuclear fraction supernatant was used for immunoprecipitation, as described above. Horseradish peroxidase-linked secondary antibodies (GE Healthcare, Little Chalfont, UK) and ECL (GE Healthcare) or Supersignal (Pierce) were used to detect immune complexes. Protein expression levels were quantified using ImageJ software v1.74c (NIH, Bethesda, MD).

To determine whether LSD1 was capable of forming dimers LSD1-FLAG and LSD1-MYC constructs were lentivirally expressed in THP1-rtTA and cells were selected with puromycin and blasticidin. Following induction with doxycycline for 24hr cells were lysed in TNN Buffer and lysate was incubated with the appropriate antibody pre-bound to Protein G–Sepharose (Sigma Aldrich) for two hours. Immune complexes were pulled down and washed four times with TNN buffer, eluted and separated by SDS-PAGE.

**LSD1 *in vitro* demethylation assay**

To immunoprecipitate $LSD1_{wt}$ or $LSD1_{K661A}$, cells were lysed in high salt lysis buffer and immune complexes were prepared as described above. Complexes were washed four times with high salt lysis buffer and once in TNN buffer. The *in vitro* demethylation assay was performed as previously described (Lynch et al., 2013). Recombinant human LSD1 was from AMS Biotechnology (Abingdon, UK). Peptides were: SNAG domain (MPRSFLVKSK) (Genscript, Piscataway, NJ) or myelin basic peptide (aa 87-99) control (VHFFLNIVTPRTP) (Bio-Techne, Minneapolis, MN).

**Protein purification and mass spectrometry**

Recombinant GST-$LSD1_{wt}$ or GST-$LSD1_{K661A}$ were purified as described (Bultsma et al., 2010). Following purification GST-$LSD1_{wt}$ and GST-$LSD1_{K661A}$ were treated with 250nM OG86 for 2 hours at 25ºC in

100mM NaCl and 50mM HEPES (pH7.5). 0.1% trifluoroacetic acid (TFA) was added and applied to a C18 stage tip (Proxeon, Thermo, Hemel Hempstead, UK) and eluted with 50:50 $CH_3CN/H_2O$ containing 0.1% TFA. The eluent was analyzed by MALDI-TOF mass spectrometry (Bruker Ultraflex II TOF/TOF (Bruker, Coventry, UK) run in positive reflectron ion mode) in α-cyano-4-hydroxycinnamic acid.

**Expression constructs, lentiviral and retroviral vectors**

Lentiviral vectors (pLKO.1) targeting human (TRCN0000382379) or murine (TRCN0000071376) *Lsd1* for knockdown, or a non-targeting control (NTC) (SHC002), were from Sigma Aldrich.

To generate an expression construct for full length human LSD1 (1-852), human *LSD1* cDNA was PCR amplified from Kazusa clone KIAA0601 (ORK00571; Chiba, Japan) (NP_055828) using oligonucleotides which introduced coding sequences for a C-terminal GSG linker and Myc tag:

F: cacgaattcaccatgttatctgggaagaaggcggcag

R: cacctcgagtcacagatcctcttctgagatgagtttttgttcacccgaacccatgcttggggactgctgtgcagg

The product was sub-cloned into pGEM-T and then excised and ligated into the EcoRI and XhoI sites of $pcDNA3.1^+$. To generate a retroviral construct expressing full-length *LSD1*, LSD1-GSG-MYC cDNA was excised from $pcDNA3.1^+$ using EcoRI and XhoI and sub-cloned into pMSCV-IRES-GFP. To make the K661A mutant vector, the following primers were used in a site-directed mutagenesis reaction using pMSCV-MYC-tag-LSD1WT-IRES-GFP as the template vector:

F: tgggatttggcaaccttaacgcggtggtgttgtgttttgatc

R: gatcaaaacacaacaccaccgcgttaaggttgccaaatccca

To generate a construct encoding an N-terminal truncated form of LSD1 (172-833) for protein expression and purification, human *LSD1* cDNA was PCR amplified from Kazusa clone KIAA0601 using the following oligonucleotides:

GST LSD1 BglII F: cacagatcttcgggtgtggagggcgcagctttc

GST LSD1 EcoRI R: cacgaattcttattacatggcccccaaaaactggtctgc

The product was digested using BglII and EcoRI and ligated into the BglII and EcoRI sites of pGex4T-1. The K661A mutant vector was made using the above mentioned mutagenesis primers and pGex4T-1-GST-tag-LSD1 WT as a template.

To generate tetracycline inducible LSD1, GFI1 and GFI1 ZNF lentiviral expression constructs, fragments coding full length FLAG-LSD1, FLAG-GFI1 or FLAG-GFI1 ZNF were amplified using the $pcDNA3.1^+$ LSD1 construct or murine *Gfi1 cDNA* (a gift from Georges Lacaud, Manchester, UK) as templates and sub-cloned into pGEM-T. Sequence verified cDNAs were excised using EcoRI and XbaI

and sub-cloned into pLentiGS-minCMV-TET-puromycin. The GFI1 ZNF LSD1 and GFI1 LSD1 fusion constructs were generated by sequential ligation of PCR amplified EcoRI/SalI FLAG GFI1 ZNF or FLAG GFI1 and XhoI/XbaI LSD1 fragments assembled in pGEM-T by SalI and XhoI ligation of the fragments. Full length fusion products were excised using EcoRI and XbaI and sub-cloned into pLentiGS-minCMV-TET-puromycin. All constructs were generated using oligonucleotides which retained or introduced coding sequences for an N-terminal FLAG tag:

| Construct | Primer sequences |
|---|---|
| LSD1 | F gaattcatggattacaaggatgacgacgataagatgttatctgggaagaaggcggc<br>R tctagattacatgcttggggactgctgtg |
| GFI1 | F atagaattcatggattacaaggatgacgacgataag<br>R atatctagattatttgagtccatgctgagtctctcg |
| GFI1 ZNF | F gaattcatggattacaaggatgacgacgataagtcctacaaatgcatcaaatgc<br>R tctagattatttgagtccatgctgagtc |
| GFI1 LSD1 | F atagaattcatggattacaaggatgacgacgataag<br>R gtcgactttgagtccatgctgagtctctcg |
| GFI1 LSD1 | F ctcgagatgttatctgggaagaaggcg<br>R ctagattacatgcttggggactgctgtg |
| GFI1 ZNF LSD1 | F gaattcatggattacaaggatgacgacgataagtcctacaaatgcatcaaatgc<br>R gtcgactttgagtccatgctgagtctctcg |
| GFI1 ZNF LSD1 | F ctcgagatgttatctgggaagaaggcg<br>R tctagattacatgcttggggactgctgtg |

Red text denotes portion of the fusion amplified by the indicated primers.

To generate the RCOR1 lentiviral expression construct, RCOR1 was PCR amplified from THP1 cDNA and ligated into EcoRI and XbaI sites of pLentiGS-minCMV-TET-puromycin. The GFI1 ZNF RCOR1 fusion construct was generated from fragments coding for FLAG-GFI1 ZNF and full length RCOR1 that were PCR amplified using full length murine *Gfi1* and THP1 cDNA as templates. Fragments were sub-cloned into pGEM-T Easy and sequence verified to confirm orientation. To assemble the fusion, RCOR1 was excised using XhoI/SacI and cloned into SalI/SacI sites of linearized pGEM-T FLAG GFI1 ZNF. The assembled fusion product was excised from pGEM-T using EcoRI and XbaI and cloned into the corresponding restriction sites of pLentiGS-minCMV-TET-puromycin. Constructs were generated using oligonucleotides which introduced a coding sequence for an N-terminal FLAG tag. The GFI1 ZNF Δ1-179 RCOR1 construct was produced by site directed mutagenesis of GFI1 ZNF RCOR1 by deletion of nucleotides 1-537 of RCOR1:

| Construct | Primer sequences |
|---|---|
| RCOR1 | F gaattcatggattacaaggatgacgacgataagatgccggccatggtggagaagg<br>R tctagattaggaggcagatgcatatctgac |
| GFI1 ZNF RCOR1 | F atagaattcatggattacaaggatgacgacgataag<br>R gtcgactttgagtccatgctgagtctctcg |
| GFI1 ZNF RCOR1 | F ctcgagatgccggccatggtggagaagg<br>R tctagattaggaggcagatgcatatctgac |
| GFI1 ZNF Δ1-179 RCOR1 | F gcatggactcaaagtcgagaagtcattggctgatttgc<br>R gcaaatcagccaatgacttctcgactttgagtccatgc |

Red text denotes portion of the fusion amplified by the indicated primers.

To create FLAG HDAC1, human HDAC1 cDNA was PCR amplified from FLAG HDAC1 pcDNA3.1- (a gift from Nullin Divecha, Manchester, UK) and cloned into EcoRI/XbaI sites of pLentiGS-minCMV-TET-puromycin. The GFI1 ZNF-HDAC1 fusion construct was generated by sequential ligation of PCR amplified EcoRI/SalI FLAG GFI1 ZNF and XhoI/XbaI HDAC1 fragments assembled in pGEM-T by SalI and XhoI ligation. Full length fusion products were excised using EcoRI and XbaI and sub-cloned into pLentiGS-minCMV-TET-puromycin. FLAG GFI1 ZNF RCOR1 (1-179) HDAC1 and FLAG RCOR1 (1-179) HDAC1 fusion constructs were generated by ligation of BclI/XbaI HDAC1 cDNAs into FLAG GFI1 ZNF RCOR1 pLentiGS-minCMV-TET-puromycin and FLAG RCOR1 pLentiGS-minCMV-TET-puromycin constructs following insertion of a BamHI site by site directed mutagenesis at nucleotide positions 564-569 of RCOR1.

| Construct | Primer sequences |
|---|---|
| HDAC1 | F atagaattcatggattacaaggatgacgacgataag<br>R atatctagattaggccaacttgacctcctcctt |
| GFI1 ZNF HDAC1 | F atagaattcatggattacaaggatgacgacgataag<br>R gtcgactttgagtccatgctgagtctctcg |
| GFI1 ZNF HDAC1 | F ctcgagatggcgcagacgcagggcac<br>R atatctagattaggccaacttgacctcctcctt |
| RCOR1 (1-179) BamH1 SDM HDAC1 | F tcttctggcataaacataatatcgaaggatccaagtcattggctgatttgc<br>R gcaaatcagccaatgacttggatccttcgatattatgtttatgccagaaga |
| RCOR1 (1-179) HDAC1 | F tgatcaatggcgcagacgcagggcac<br>R tctagattaggccaacttgacctcctccttgacc |
| GFI1 ZNF RCOR1 (1-179) BamH1 SDM HDAC1 | F tcttctggcataaacataatatcgaaggatccaagtcattggctgatttgc<br>R gcaaatcagccaatgacttggatccttcgatattatgtttatgccagaaga |
| GFI1 ZNF RCOR1 (1-179) HDAC1 | F tgatcaatggcgcagacgcagggcac<br>R tctagattaggccaacttgacctcctccttgacc |

Red text denotes portion of the fusion amplified by the indicated primers.

To generate the pLentiGS-minCMV-TET-puromycin vector, sequence coding for the SV40 promoter and blasticidin selection marker were excised from pLentiGS-minCMV-TET-blasticidin using XbaI and SalI. This was replaced by an SV40 puromycin fragment excised from EF1α pLentiGS-puromycin (Huang et al., 2014), also using XbaI and SalI.

GFI1 ZNF LSD1 Tower domain mutants were generated by individual or sequential rounds of site directed mutagenesis of FLAG GFI1 ZNF LSD1 in the pLentiGS-minCMV-TET-puromycin lentiviral plasmid. Oligonucleotides used for the mutagenesis PCR reactions were:

| GFI1 ZNF LSD1 Tower domain mutant | Primer sequences |
|---|---|
| K421>D K424>D | F tcttccaatgttcaatctgctcatcgtcgacatgatcctcttgtaactgaatgacaacttcc<br>R ggaagttgtcattcagttacaagaggatcatgtcgacgatgagcagattgaacattggaaga |
| K452>D | F gtattgctgatggagttctttaattttctcatccaaatttaccatcttattaagaagttcttt<br>R aaagaacttcttaataagatggtaaatttggatgagaaaattaaagaactccatcagcaatac |
| D486>K | F cttgcatagggcggtcagtttcctgtgtttgcttttcac<br>R gtgaaaagcaaacacaggaaactgaccgccctatgcaag |
| D495>K | F ccttgtgtttcagctaattccttatattccttgcatagggcgg<br>R ccgccctatgcaaggaatataaggaattagctgaaacacaagg |

To generate lentiviral *GFI1* knockdown constructs, pLKO.1 Puro was digested with AgeI and EcoRI and ligated with HPLC purified oligonucleotides previously annealed by incubating at 98$^{o}$C for 5 mins, and slowly cooling to room temperature. Oligonucleotide sequences were:

KD#1

F ccggccagactattccctccgtttactcgagtaaacggagggaatagtctggtttttg

R aattcaaaaaccagactattccctccgtttactcgagtaaacggagggaatagtctgg

KD#2

F ccggcgacctctgtgggaagggtttctcgagaaacccttcccacagaggtcgtttttg

R aattcaaaaacgacctctgtgggaagggtttctcgagaaacccttcccacagaggtcg

**Supplemental references**


Andrews S. 2010. FastQC: http://www.bioinformatics.babraham.ac.uk/projects/fastqc/

Buenrostro, J. D., Giresi, P. G., Zaba, L. C., Chang, H. Y., and Greenleaf, W. J. (2013). Transposition of native chromatin for fast and sensitive epigenomic profiling of open chromatin, DNA-binding proteins and nucleosome position. Nature Methods *10*, 1213-8.

Bultsma, Y., Keune, W.J., and Divecha, N. (2010). PIP4Kbeta interacts with and modulates nuclear localization of the high-activity PtdIns5P-4-kinase isoform PIP4Kalpha. Biochem J. 430, 223-35.

David, M., Dzamba, M., Lister, D., Ilie, L., and Brudno, M. (2011). SHRiMP2: Sensitive yet Practical Short Read Mapping. Bioinformatics *27*, 1011-1012.

Gentleman, R., Carey, V., Bates, D., Bolstad, B., Dettling, M., Dudoit, S., Ellis, B., Gautier, L., Ge, Y., Gentry, J., et al. (2004). Bioconductor: open software development for computational biology and bioinformatics. Genome Biology *5*, R80.

Huang, X., Spencer, G. J., Lynch, J. T., Ciceri, F., Somerville, T. D., and Somervaille, T. C. (2014). Enhancers of Polycomb EPC1 and EPC2 sustain the oncogenic potential of MLL leukemia stem cells. Leukemia *28*, 1081-1091.

Karasuyama, H., and Melchers, F. (1988). Establishment of mouse cell lines which constitutively secrete large quantities of interleukin 2, 3, 4 or 5, using modified cDNA expression vectors. Eur J Immunol 18, 97-104.

Langmead, B., Trapnell C., Pop M., and Salzberg S. L. (2009). Ultrafast and memory-efficient alignment of short DNA sequences to the human genome. Genome Biology *10*, R25.

Lawrence, M., Huber, W., Pages, H., Aboyoun, P., Carlson, M., Gentelman, R., Morgan, M.T.,Carey, V.J. (2013). Software for Computing and Annotating Genomic Ranges. PLoS Comput Biol *9*: e1003118.

Lee, T. I., Johnstone, S. E., and Young R. A. (2006). Chromatin immunoprecipitaion and microarray-based analysis of protein location. Nature Protocols, *1*, 729-748.

Li, H. and Durbin, R. (2009). Fast and accurate short read alignment with Burrows-Wheeler Transform. Bioinformatics *25*, 1754-60.

Li, H., Handsaker, B., Wysoker, A., Fennell, T., Ruan, J., Homer, N., Marth, G., Abecasis, G., and Durbin, R., for the 1000 Genome Project Data Processing subgroup (2009). The Sequence Alignment/Map format and SAMtools. Bioinformatics *25*, 2078-9.

Martin, M. (2012). Cutadapt removes adapter sequences from high-throughput sequencing reads. Bioinformatics in Action *17*, 10-12.

Spyrou, C., Stark, R., Lynch, A.G. and Tavaré, S. (2009). BayesPeak: Bayesian analysis of ChIP-seq data. BMC Bioinformatics *10*, 299.

Quinlan, A.R. and Hall, I.M. (2010). BEDTools: a flexible suite of utilities for comparing genomic features. Bioinformatics *26* :841-2.

Subramanian, A., Tamayo, P., Mootha, V. K., Mukherjee, S., Ebert, B. L., Gillette, M. A., Paulovich, A., Pomeroy, S. L., Golub, T. R., Lander, E. S., and Mesirov, J. P. (2005). Gene set enrichment analysis: a knowledge-based approach for interpreting genome-wide expression profiles. Proc Natl Acad Sci U S A *102*, 15545-15550.

Wang, C., Lee, J.E., Lai, B., Macfarlan, T.S., Xu, S., Zhuang, L., Liu C., Peng and W., and Ge, K. (2016). Enhancer priming by H3K4 methyltransferase MLL4 controls cell fate transition. PNAS 113,11871-11876.

Yates T., Okoniewski M.J., and Miller C.J. (2007). X:Map: annotation and visualization of genome structure for Affymetrix exon array analysis. Nucleic Acids Res. *36*, D780-D786.

Yu, G., Wang, L., and He, Q. (2015). ChIPseeker: an R/Bioconductor package for ChIP peak annotation, comparison and visualization. Bioinformatics*, 31*, 2382-2383.

Zhang, Y., Liu, T., Meyer, C. A., Eckhoute, J., Johnson, D. S., Bernstein, B. E., Nusbaum, C., Myers, R. M., Brown, M., Li, W., et al., (2008). Model-based analysis of ChIP-Seq (MACS). Genome Biology *9*, R137.
